# Longer Prehospitalization and Preintubation Periods in Intubated Non-survivors and ECMO Patients With COVID-19: A Systematic Review and Meta-Analysis

**DOI:** 10.3389/fmed.2021.727101

**Published:** 2021-10-15

**Authors:** Kenji Funakoshi, Takayoshi Morita, Atsushi Kumanogoh

**Affiliations:** ^1^Department of Respiratory Medicine and Clinical Immunology, Graduate School of Medicine, Osaka University, Suita, Japan; ^2^Department of Immunopathology, WPI, Immunology Frontier Research Center (iFReC), Osaka University, Suita, Japan; ^3^Integrated Frontier Research for Medical Science Division, Institute for Open and Transdisciplinary Research Initiatives (OTRI), Osaka University, Suita, Japan; ^4^Center for Infectious Diseases for Education and Research (CiDER), Osaka University, Suita, Japan

**Keywords:** COVID-19, clinical course, invasive mechanical ventilation, extracorporeal membrane oxygenation, meta-analysis

## Abstract

**Purpose:** There is no clear consensus on the clinical course of critical COVID-19 patients. We examined the clinical course among intubated survivors, non-survivors, and extracorporeal membrane oxygenation (ECMO) patients to reveal the standard clinical course and the difference among critical COVID-19 patients.

**Methods:** In this systematic review and meta-analysis, we searched PubMed, Web of Science, and Scopus for original studies published until December 11, 2020, including case accumulation and clinical course reporting. Pregnant patients and children were excluded. We followed PRISMA guidelines and registered them with PROSPERO (CRD42021235534).

**Results:** Of the 11,716 studies identified, 94 met the selection criteria, and 2,549 cases were included in this meta-analysis. The times from intubation to extubation and death were 12.07 days (95% confidence interval 9.80–14.33 days) and 10.14 days (8.18–12.10 days), respectively, and the ECMO duration was 14.72 days (10.57–18.87 days). The time from symptom onset to hospitalization (prehospitalization period) of intubated survivors, non-survivors, and ECMO patients was 6.15 (4.61–7.69 days), 6.45 (4.55–8.34 days), and 7.15 days (6.48–7.81 days), and that from symptom onset to intubation (preintubation period) was 8.58 (7.36–9.80 days), 9.14 (7.26–11.01 days), and 10.54 days (9.18–11.90 days), respectively. Sensitivity analysis showed that the time from intubation to extubation and death was longer in the US and Europe than in East Asia.

**Conclusion:** For COVID-19, we hypothesize that prehospitalization and preintubation periods are longer in intubated non-survivors and ECMO patients than in intubated survivors. These periods may serve as a predictor of disease severity or death and support therapeutic strategy determination.

## Introduction

Coronavirus disease 2019 (COVID-19) is a pandemic caused by severe acute respiratory syndrome coronavirus 2 (SARS-CoV-2) and was first reported in Wuhan, China, in December 2019 (1). As of August 2021, COVID-19 had spread to 223 countries, areas, or territories, and the global cumulative case numbers have reached 197 million. Over 4.2 million COVID-19 patients have died since the start of the pandemic (2), even though every government has taken aggressive preventive measures such as lockdown (3), universal masking (4), and social distancing (4). The hospitalization rate of COVID-19 is reportedly 14% (almost 10 times higher than influenza) (5–7). Moreover, up to 26.1% of hospitalized COVID-19 patients are admitted to the intensive care unit (ICU) (8). Therefore, COVID-19 has placed an unprecedented burden on the ICU, and in some regions, ICU capacity exceeds 100% with only COVID-19 patients because of the astonishing number, high rate of ICU admission, and long clinical course (9). Furthermore, 71–88% of COVID-19 patients in the ICU need intubation (2.45–4.01 times higher than influenza) (10–14), and 3–27.2% of intubated COVID-19 patients require ECMO (10, 15). Overall, the high occupancy rate of hospital beds and ICUs by COVID-19 patients is a serious problem worldwide.

The clinical course of patients with severe COVID-19 from symptom onset to clinical events is highly informative when considering prognosis, therapeutic strategy, ICU bed management, and medical economy. Nevertheless, comparing each patient's clinical course with the standard clinical course of COVID-19 is difficult because there is no consensus to date regarding the standard clinical course. For example, the duration of intubation has been reported to be 10–16 days (16, 17), yet both the patients' backgrounds and regions where the studies were conducted differed in these reports. Moreover, known risk factors for COVID-19 mortality include age (18), sex (19), comorbidities (19), and blood counts (absolute lymphocyte number and CRP) (20); however, few articles have assessed differences in the clinical course between intubated survivors, non-survivors, and ECMO patients.

In this study, we conducted a systematic review and meta-analysis of the clinical course, i.e., time (days) from symptom onset, hospitalization, intubation, and ECMO initiation to each clinical event in critical COVID-19 patients. We also assessed the difference in the clinical course between intubated survivors, non-survivors, and ECMO patients with COVID-19 to reveal whether the clinical course is a prognostic factor. Finally, we conducted sensitivity analysis to assess factors (patient background and region) that may influence the time from intubation to extubation or death.

## Methods

### Search Strategy and Selection Criteria

This meta-analysis was performed following the Preferred Reporting Items for Systematic Reviews and Meta-analyses (PRISMA) statement (Supplementary Table 1) (21). This study searched for articles documenting the clinical course in critical COVID-19 patients: the time (days) from symptom onset to hospitalization (prehospitalization period) to intubation (preintubation period) and to ECMO initiation (pre-ECMO period); the time from hospitalization to intubation (hospitalization-intubation period) and to ECMO initiation (hospitalization-ECMO period), discharge (hospitalization-discharge period), and death (hospitalization-death period); the time from hospitalization to death (hospitalization-death period); the time from intubation to extubation (intubation period), to ECMO (intubation-ECMO period), and to death (intubation-death period); and the time from ECMO initiation to decannulation (ECMO period) and to death (ECMO-death period). Three sources, namely, PubMed, Web of Science, and Scopus, were searched [(COVID-19) OR (SARS-CoV-2) AND (intensive care unit) OR (acute respiratory distress syndrome) OR (mechanical ventilation) OR (extracorporeal membrane oxygenation)], with no language restriction. The searches were performed to identify articles published until December 11th, 2020, when the SARS-CoV-2 vaccine was first approved in the world, including “online first” articles, published until December 11, 2020, when the SARS-CoV-2 vaccine was first approved. The last searches were performed on June 26, 2021.

The inclusion criteria were studies of human subjects, case accumulations, a title or abstract consisting of the clinical course of intubated survivors, non-survivors, and/or ECMO patients with COVID-19, and a link from the search site to the full text (PDF or website) of the article. In this study, “survivors” referred to extubated patients who had not died during the study period. This study excluded studies involving children (under 18 years old) and pregnant women and non-English articles; a case report was also excluded because properly calculating the average value and standard deviation (SD) was difficult. Redundancies between the search sites were eliminated, i.e., individual studies were counted only once in this analysis.

### Data Extraction and Quality Assessment

Data were extracted from all studies included in this analysis (author, year of publication, country where the study was conducted, number of patients, age, percentage of males, comorbidities, and treatment); the details are provided in Table 1. The average number of days and SD showing each clinical course or the median number of days and interquartile range (IQR) and/or range were extracted. The average number of days and SD were calculated from the median and IQR or range data using the reported methods if only the median and IQR or range were given in the study (22).

**Table 1:** Background of critical COVID-19 patients.

**IMV/ECMO**	**Study**	**Sample size**	**Location of study**	**Age mean (SD) or**	**Male**	**HTN**	**DM**	**Reported treatment (%)**						**Risk of bias**
				**Median (IQR)**				**GC**	**TCZ/SAR**	**HCQ**	**REM**	**L/R**	**Others**	
IMV	Abe et al.	2	Japan	64 (4)	50	0	100	ND	100	ND	ND	ND	IVIG (100)	5
IMV	Argenziano et al.	152	US	ND	ND	ND	ND	ND	ND	ND	ND	ND	ND	8
IMV	Barrasa et al.	20	Spain	ND	ND	ND	ND	ND	ND	ND	ND	ND	ND	8
IMV	Beigmohammadi et al.	7	Iran	66.67 (11.47)	71.43	57.14	14.23	ND	ND	100	14.29	ND	ND	5
IMV	Bhatraju et al.	18	US	ND	ND	ND	ND	ND	ND	ND	ND	ND	ND	6
IMV	Cauchois et al.	5	France	ND	ND	ND	ND	ND	ND	ND	ND	ND	Anakinra (40)	7
IMV	Chen et al.	2	China	65 (2)	100	ND	ND	ND	ND	ND	ND	ND	ND	6
IMV	Christie 3rd et al.	2	US	75 (4.11)	50	100	0	ND	ND	ND	ND	ND	ND	6
IMV	Cummings et al.	163	US	ND	ND	ND	ND	ND	ND	ND	ND	ND	ND	7
IMV	Dai et al.	5	China	ND	ND	ND	ND	ND	ND	ND	ND	ND	ND	8
IMV	Dastan et al.	6	Iran	ND	ND	ND	ND	ND	ND	ND	ND	ND	ND	9
IMV	De Luca et al.	3	Italy	ND	ND	ND	ND	ND	ND	ND	ND	ND	GM-CSF blockade (100)	9
IMV	Dogan et al.	4	Turkey	45.25 (13.94)	75	50	33.33	ND	ND	100	ND	25	Plasmapheresis (100)	6
IMV	Elder et al.	3	US	73.33 (3.77)	66.67	ND	ND	ND	ND	ND	ND	ND	ND	5
IMV	Falces-Romero et al.	5	Spain	66.6 (8.36)	60	0	100	100	20	100	0	20	ND	5
IMV	Flikweert et al.	7	Netherlands	73 (7.48)	71.43	28.57	14.23	57.14	ND	85.71	ND	ND	Heparin (100)	5
IMV	Gavin et al.	53	US	ND	67.92	73.58	45.28	ND	ND	ND	ND	ND	ND	8
IMV	Grasselli et al.	836	Italy	68 (62–73)	83.73	59.81	21.77	ND	ND	ND	ND	ND	ND	8
IMV	Grein et al.	19	US	ND	ND	ND	ND	ND	ND	ND	100	ND	ND	7
IMV	Halvatsiotis et al.	26	Greece	65 (53–70)	80.77	46.15	30.77	ND	ND	ND	ND	ND	ND	8
IMV	Hernandez-Romieu et al.	63	US	ND	ND	ND	ND	ND	ND	ND	ND	ND	ND	7
IMV	Kato et al.	7	Japan	ND	ND	ND	ND	ND	ND	ND	ND	ND	ND	7
IMV	Ketcham et al.	2	US	ND	ND	ND	ND	ND	ND	ND	ND	ND	ND	7
IMV	Kewan et al.	2	US	ND	ND	ND	ND	ND	0	ND	ND	ND	ND	7
IMV	Khullar et al.	17	US	57 (Range 25, 75)	64.71	47.06	41.18	ND	ND	ND	ND	ND	ND	8
IMV	Konopka et al.	3	US	54 (16.5)	66.67	33.33	100	33.33	33.33	66.67	ND	ND	ND	7
IMV	Krishnan et al.	92	US	71 (10)	64.13	40.22	25	58.70	11.96	93.48	ND	ND	ND	8
IMV	Kristinsson et al.	15	Iceland	ND	ND	ND	ND	ND	ND	ND	ND	ND	ND	7
IMV	Lê et al.	2	France	ND	ND	ND	ND	ND	ND	ND	ND	ND	ND	6
IMV	LeBrun et al.	3	US	89 (3.74)	66.67	100	66.67	ND	ND	ND	ND	ND	ND	7
IMV	Lechien et al.	15	Italy	66.8 (11.97)	93.33	ND	ND	ND	ND	ND	ND	ND	ND	8
IMV	Lee et al.	2	Singapore	62.5 (8.5)	100	ND	ND	ND	ND	ND	ND	ND	ND	7
IMV	Liu et al.	42	China	ND	ND	ND	ND	ND	ND	ND	ND	ND	ND	8
IMV	Lowe et al.	2	US	59.5 (1.5)	100	50	50	ND	ND	100	ND	ND	ND	6
IMV	Maritati et al.	2	Italy	67.5 (4.5)	50	100	0	100	100	100	ND	50	ND	5
IMV	Morassi et al.	4	Italy	63.25 (7.36)	100	50	25	ND	ND	ND	ND	ND	ND	5
IMV	Morillas et al.	3	US	62.67 (10.96)	33.33	66.67	33.33	66.67	100	100	ND	33.3	ND	7
IMV	Navarro-Millán et al.	5	US	61.4 (10.13)	100	80	60	100	20	ND	0	ND	Anakinra (100)	6
IMV	Novelli et al.	3	Italy	ND	ND	ND	ND	ND	ND	ND	ND	ND	ND	8
IMV	Pan et al.	3	China	ND	ND	ND	ND	ND	ND	ND	ND	ND	ND	6
IMV	Peng et al.	7	China	56.43 (11.15)	42.86	28.57	14.29	100	ND	ND	ND	100	ND	6
IMV	Plotnikow et al.	37	Argentina	ND	81.8	32.43	29.73	ND	ND	ND	ND	ND	ND	8
IMV	Radnis et al.	2	US	38 (6)	0	0	0	ND	ND	ND	ND	ND	ND	5
IMV	Riker et al.	2	US	72 (2)	100	100	0	ND	ND	50	ND	ND	ND	5
IMV	Rizo-Téllez et al.	10	Mexico	ND	ND	ND	ND	ND	ND	ND	ND	ND	ND	7
IMV	Sakr et al.	2	Germany	57.5 (8.5)	100	50	50	ND	ND	ND	ND	ND	Heparin (50), Enoxaparin (50)	5
IMV	Schaefer et al.	5	US	66 (8.80)	60	80	80	ND	ND	40	20	ND	ND	5
IMV	Shen et al.	3	China	50.67 (12.47)	33.33	33.33	0	100	ND	ND	ND	100	ND	7
IMV	Singh et al.	4	US	52.25 (20.56)	100	ND	ND	ND	100	ND	ND	ND	CAP-1002 (100)	6
IMV	So et al.	7	Japan	62.23 (12.48)	57.14	42.86	42.86	100	ND	ND	ND	ND	Heparin (100)	6
IMV	Søvik et al.	4	Norway	70 [Range 62–75]	100	25	ND	ND	ND	ND	ND	ND	ND	7
IMV	Stony Brook COVID-19 Research Consortium	87	US	ND	ND	ND	ND	ND	ND	ND	ND	ND	ND	7
IMV	Wali et al.	3	France	63.33 (4.71)	100	0	33.33	ND	ND	ND	ND	ND	ND	6
IMV	Wang et al.	97	China	70 (62–77)	76.29	71.13	30.93	ND	ND	ND	ND	ND	ND	7
IMV	Wang et al.	2	China	66 (3)	100	ND	ND	ND	ND	ND	ND	ND	ND	5
IMV	Weiskopf et al.	5	US	60.6 (3.01)	60	ND	ND	ND	ND	60	ND	60	ND	6
IMV	Wilk et al.	2	US	49 (15)	100	ND	ND	ND	ND	ND	ND	ND	ND	7
IMV	Zhang et al.	12	China	71.33 (7.70)	50	58.33	16.67	ND	ND	ND	ND	ND	ND	8
IMV	Ziehr et al.	41	US	ND	ND	ND	ND	ND	ND	ND	ND	ND	ND	7
ECMO	Akhtar et al.	18	UK	47.3 (9.8)	88.89	55.56	55.56	ND	ND	ND	ND	ND	ND	7
ECMO	Alnababteh et al.	13	US	44.54 (9.49)	61.54	38.46	30.77	30.77	69.23	76.92	ND	ND	Anticoagulation (92.31)	8
ECMO	Beyls et al.	12	France	62 (56-66)	83.33	ND	ND	ND	ND	ND	ND	ND	ND	5
ECMO	Charlton et al.	16	UK	47.0 (8.4)	75	12.5	6.25	ND	ND	ND	ND	ND	ND	7
ECMO	Dastan et al.	3	Iran	ND	ND	ND	ND	ND	ND	ND	ND	ND	ND	9
ECMO	Falcoz et al.	17	France	ND	94.12	52.94	17.65	47.06	ND	47.06	ND	94	ND	7
ECMO	Goursaud et al.	2	France	58.5 (5.5)	ND	ND	ND	100	ND	ND	ND	ND	ND	5
ECMO	Grein et al.	5	US	ND	ND	ND	ND	ND	ND	ND	100	ND	ND	7
ECMO	Guihaire et al.	24	France	ND	83.33	20.83	20.83	ND	ND	ND	ND	ND	ND	6
ECMO	Guo et al.	7	China	69.29 (6.98)	85.71	57.14	28.57	ND	ND	ND	ND	ND	ND	5
ECMO	Heman-Ackah et al.	2	US	52 (6)	50	50	50	ND	ND	ND	ND	ND	ND	5
ECMO	Huette et al.	12	France	ND	ND	ND	ND	ND	ND	ND	ND	ND	ND	6
ECMO	Jäckel et al.	15	Germany	60.8 (54.1–67.0)	73.33	33.33	13.33	ND	ND	ND	ND	ND	ND	8
ECMO	Jacobs et al.	32	US	52.41 (12.49)	68.75	ND	34.38	15.63	18.75	3.13	ND	ND	Anti-viral therapy (18.75)	8
ECMO	Kon et al.	27	US	40 (30.5–47)	85.19	18.52	14.81	ND	ND	ND	ND	ND	ND	7
ECMO	Le Breton et al.	13	France	49.31 (7.45)	76.9	30.77	23.08	92.03	46.15	38.46	ND	ND	ND	6
ECMO	Li et al.	7	China	69.86 (7.57)	71.43	57.14	28.57	ND	ND	ND	ND	ND	ND	6
ECMO	Liu et al.	4	China	ND	ND	ND	ND	ND	ND	ND	ND	ND	ND	8
ECMO	Liu et al.	6	China	ND	ND	ND	ND	100	ND	ND	ND	100	Arbidol (100)	8
ECMO	Loforte et al.	4	Italy	49 (8.75)	100	ND	ND	ND	100	100	ND	66.67	ND	6
ECMO	Matsunaga et al.	31	Japan	ND	ND	ND	ND	ND	ND	ND	ND	ND	ND	7
ECMO	Miike et al.	3	Japan	ND	ND	ND	ND	ND	ND	ND	ND	ND	ND	6
ECMO	Mustafa et al.	40	US	48.4 (1.5)	75	57.5	25	ND	ND	ND	ND	ND	ND	6
ECMO	Osho et al.	6	US	47 (43–53)	83.33	50	66.67	ND	50	100	33.33	16.67	ND	7
ECMO	Ronit et al.	2	Denmark	52.5 (12.5)	50	0	0	ND	ND	ND	ND	ND	ND	5
ECMO	Schmidt et al.	83	France	49 (41–56)	73.49	38.55	31.33	14.46	9.64	19.28	9.64	22.89	ND	8
ECMO	Shih et al.	37	US	51 (40–59)	72.97	67.57	51.35	70.27	65.57	45.95	54.05	ND	Convalescent plasma (43.24)	7
ECMO	Sultan et al.	10	US	ND	70	ND	ND	40	30	100	40	ND	ND	6
ECMO	Usman et al.	10	US	50.7 (47.5–58.8)	70	50	ND	50	60	90	20	0	ND	7
ECMO	Xu et al.	17	China	ND	ND	ND	ND	ND	ND	ND	ND	ND	ND	7
ECMO	Xuan et al.	5	China	61.6 (9.18)	ND	80	60	80	ND	ND	ND	ND	IVIG (40)	5
ECMO	Yang et al.	21	China	58.50 (42.75–67.25)	57.14	ND	ND	ND	ND	ND	ND	ND	ND	8
ECMO	Zayat et al.	17	Germany	57.0 (53.0, 62.0)	64.71	35.29	35.29	ND	ND	ND	ND	ND	ND	8
ECMO	Zeng et al.	12	China	50.9 (13.5)	91.67	8.33	8.33	83.33	ND	ND	ND	ND	Anti-viral therapy (100)	6
ECMO	Zeng et al.	2	China	64.5 (1.5)	100	ND	ND	ND	ND	ND	ND	ND	ND	8
ECMO	Zhang et al.	43	UK	46 (35.5–52.5)	76.74	23.26	18.60	ND	ND	4.65	9.30	ND	Anakinra (23.26)	8
ECMO	Zhang et al.	3	US	55.67 (11.73)	ND	ND	ND	ND	ND	ND	ND	ND	ND	7
ECMO	Zheng et al.	11	China	ND	ND	ND	ND	ND	ND	ND	ND	ND	ND	8

*The total score was calculated based on the study quality assessment tools from the NHLBI*.

*IMV, invasive mechanical ventilation; ECMO, extracorporeal membrane oxygenation; HTN, hypertension; DM, diabetes mellitus; GC, glucocorticoid; TCZ/SAR, tocilizumab/sarilumab; HCQ, hydroxychloroquine; REM, remdesivir; L/R, lopinavir-ritonavir; IVIG, intravenous immunoglobulin; NR, not reported*.

Two authors (K.F. and T.M.) independently assessed and selected references. In cases of inconsistent results, a third author (A.K.) provided an opinion to resolve the issue. The quality of the selected studies was evaluated according to the study quality assessment tools (Quality Assessment Tool for Case Series Studies) from the National Heart, Lung, and Blood Institute (NHLBI) (23). The evidence level was assessed based on the Oxford Centre for Evidence-Based Medicine 2011 (24). Asymmetry in a funnel plot was employed to determine publication bias.

### Data Analysis

A meta-analysis was performed to estimate the clinical course of intubated survivors, non-survivors, and ECMO patients with COVID-19. Clinical data were analyzed using the metamean package. Outcomes are described as the mean number of days at each event, such as admission, intubation, or death from the onset of COVID-19 (baseline) and 95% confidence intervals (CIs) for each clinical course. For all outcomes, mean differences were calculated using the random-effects model (DerSimonian and Laird method) (25). *I*^2^ values of 25, 50, and 75% were defined as low, moderate, and high, respectively (26). All analyses were conducted using R version 4.0.3 (R Project for Statistical Computing) (27). Sensitivity analyses were carried out with regard to the intubation period and intubation-death period based on region (East Asia, the US, and Europe), age, sex, and comorbidities (hypertension and diabetes mellitus). Spearman's correlation coefficient was calculated in R version 4.0.3. *P* values ≤ 0.05 were considered statistically significant. This study was registered with PROSPERO (CRD42021235534).

## Results

**Figure 1:**
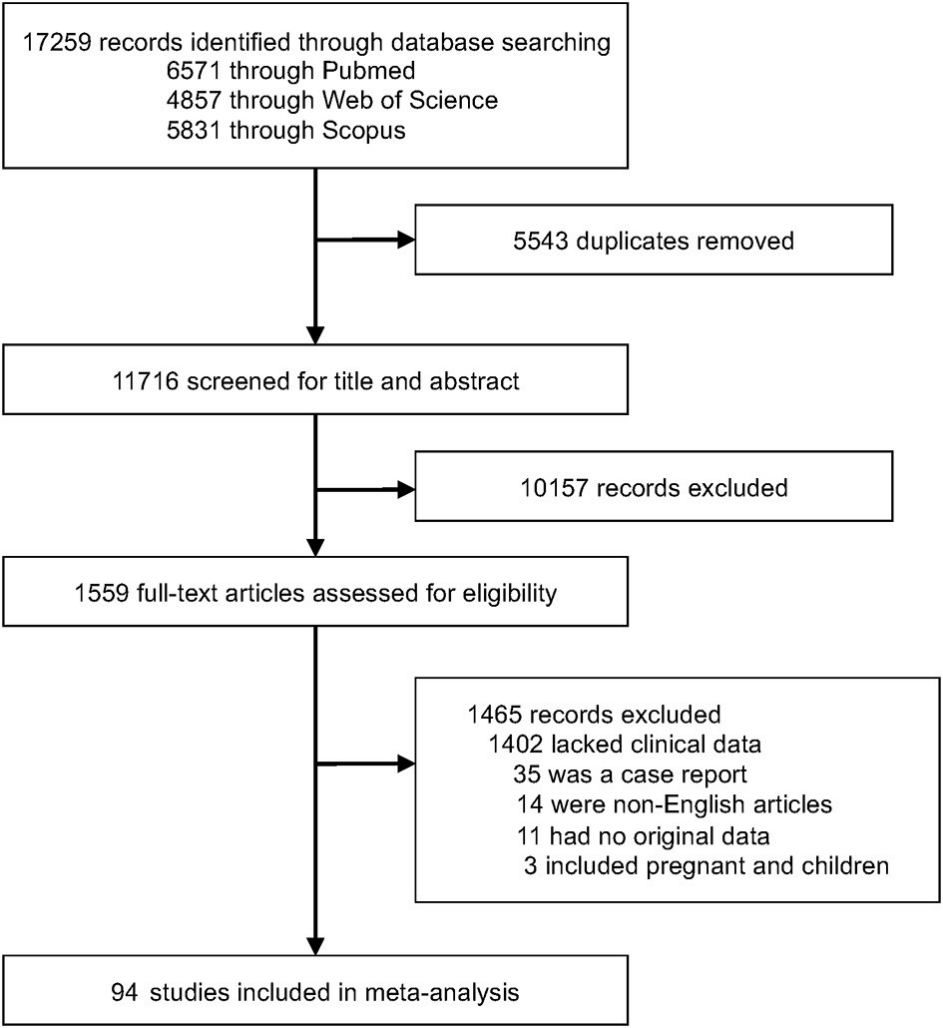
Study profile.

We identified 17,259 articles and excluded 5,543 due to duplication. We also screened 11,716 publications and identified 94 articles (15–17, 28–118), with 2,549 cases, from among 1,559 articles that underwent full-text assessment (Figure 1). Each article is summarized in Supplementary Table 1. The mean age ranged from 38 to 75 years, and the rate of male patients ranged from 0% to 100%. COVID-19 patients were reportedly treated with glucocorticoids, tocilizumab/sarilumab, remdesivir, and hydroxychloroquine; however, treatment was not described in more than 70% of the articles. There were 36 articles from the US, 19 from China, ten from France, seven from Italy, five from Japan, and a few from other countries. Despite several cohort studies, there were few intubated survivors and non-survivors, and most were case accumulations. Therefore, the risk of bias was calculated based on case accumulation. The risk of bias was more than 5 points, with 6.71 points as the average, i.e., moderate risk (Supplementary Table 2).

**Figure 2:**
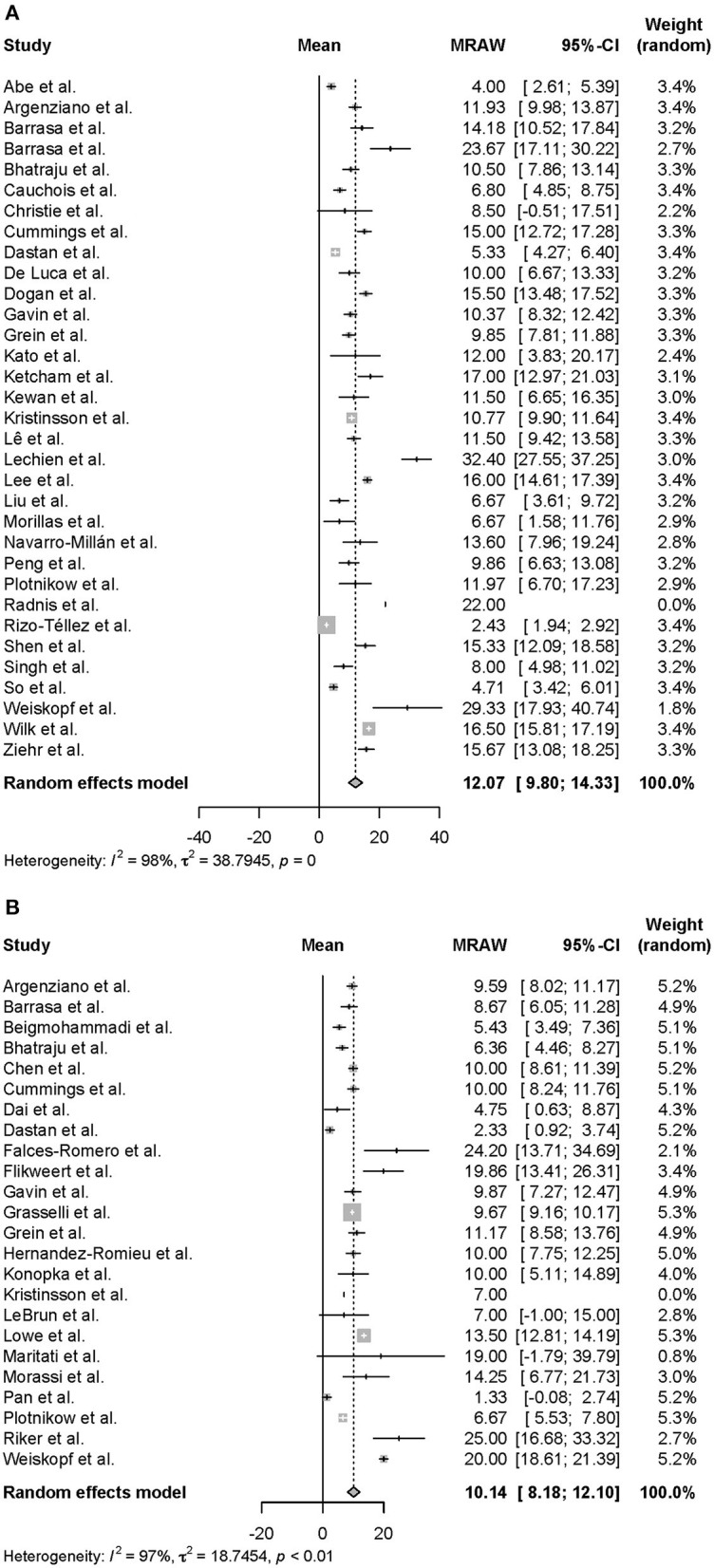
Forrest plot: a meta-analysis of the intubation period and the intubation-death period. The intubation period of intubated COVID-19 survivors **(A)** and the intubation-death period of intubated COVID-19 non-survivors **(B)** were calculated using the random effects model. MRAW, the raw data of mean; 95% CI, 95% confidence interval.

**Figure 3:**
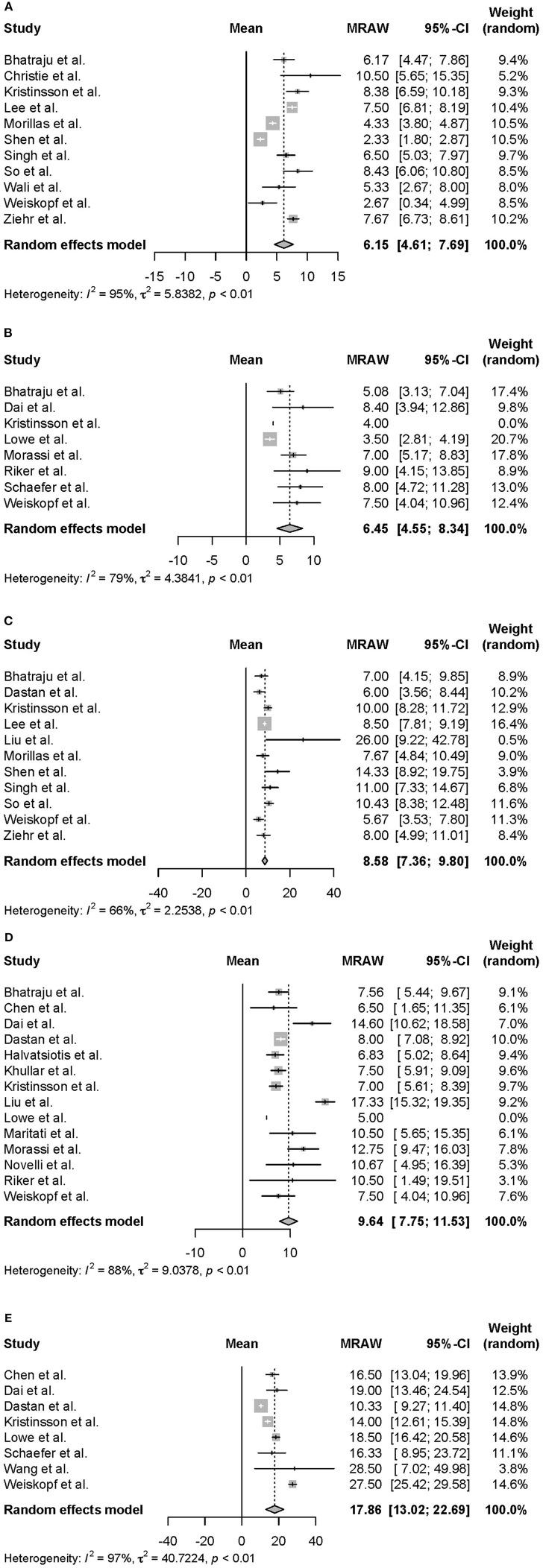
Forrest plot: a meta-analysis of the time from symptom onset to each clinical endpoint in intubated COVID-19 patients. The prehospitalization period of intubated survivors **(A)**, the prehospitalization period of intubated non-survivors **(B)**, the preintubation period of intubated survivors **(C)**, the preintubation period of intubated non-survivors **(D)**, and the symptom-death period **(E)** were calculated using the random effects model. MRAW, the raw data of mean; 95% CI, 95% confidence interval.

**Figure 4:**
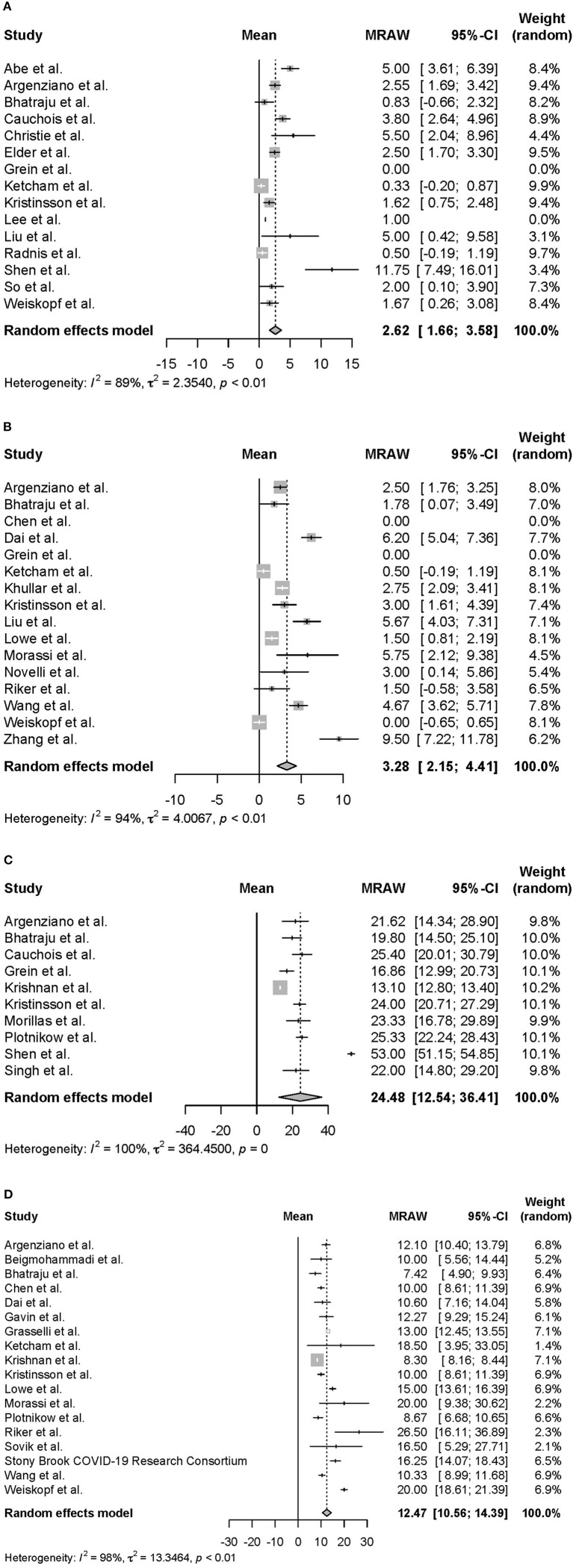
Forrest plot: a meta-analysis of the time from hospitalization onset to each clinical endpoint in intubated COVID-19 patients. The hospitalization-intubation period of intubated survivors **(A)**, the hospitalization-intubation period of intubated non-survivors **(B)**, the hospitalization-discharge period of intubated survivors **(C)**, and the hospitalization-death period of intubated non-survivors **(D)** were calculated using the random effects model. MRAW, the raw data of mean; 95% CI, 95% confidence interval.

Moreover, we conducted a meta-analysis on the clinical course of intubated survivors, non-survivors, and ECMO patients with COVID-19. First, we analyzed the intubation period and the intubation-death period of intubated survivors and non-survivors. Thirty-three reports with 325 survivors and 24 reports with 1,225 non-survivors were identified and analyzed (Figure 2). The average intubation period among intubated survivors was 12.07 days (95% CIs 9.80–14.33 days), and the average intubation-death period was 10.14 days (8.18–12.10 days). The prehospitalization periods for intubated survivors and non-survivors were 6.15 (4.61–7.69 days) and 6.45 (4.55–8.34 days) days, respectively, and the preintubation periods were 8.58 days (7.36–9.80 days) and 9.64 days (7.75–11.53 days), respectively. A symptom-death period of 17.86 days (13.02–22.69 days) was calculated (Figure 3). Additionally, the hospitalization-intubation period among intubated survivors and non-survivors was 2.62 days (1.66–3.58 days) and 3.28 days (2.15–4.41 days), respectively; the hospitalization-discharge and hospitalization-death periods were 24.48 days (12.54–36.41 days) and 12.47 days (10.56–14.39 days), respectively (Figure 4). Funnel plots are illustrated in Supplementary Figure 1.

Regarding the clinical course of those treated with ECMO, the ECMO period of both survivors and non-survivors and the ECMO-death period were 14.72 days (10.57–18.87 days) and 21.05 days (12.04–30.07 days), respectively (Supplementary Figure 2). For ECMO patients, the prehospitalization, preintubation, and pre-ECMO periods were 7.15 (6.48–7.81 days), 10.54 (9.18–11.90 days), and 14.80 (13.29–16.31 days) days, respectively, and the hospitalization-intubation, hospitalization-ECMO, and intubation-ECMO periods were 3.39 (2.08–4.69 days), 5.97 (3.91–8.02 days), and 4.57 (3.59–5.54 days) days, respectively (data not shown).

**Figure 5:**
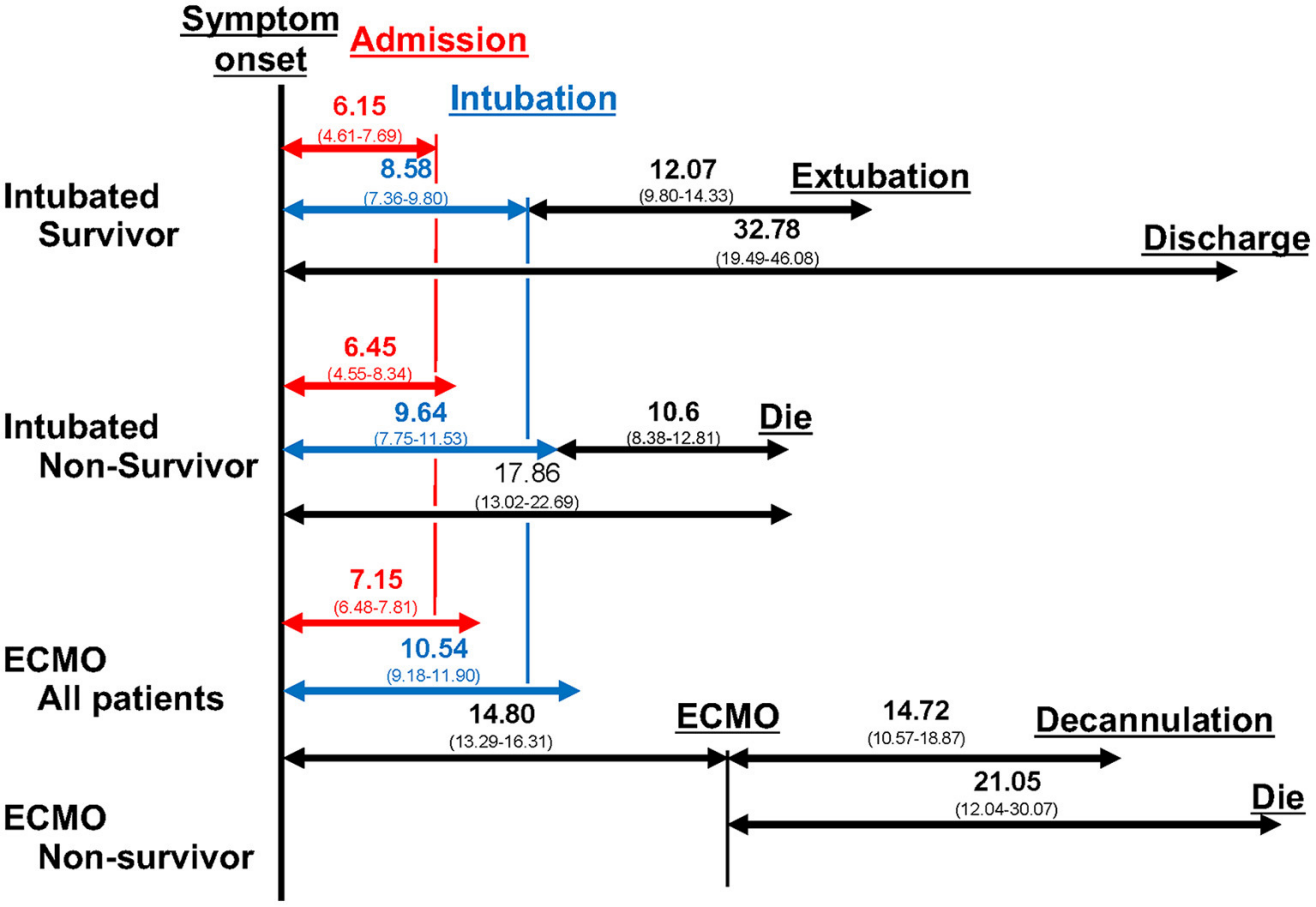
Clinical course. The duration from symptoms to each clinical event, from intubation to each clinical event, and from ECMO to each clinical event are shown. The number is the mean number of days, and the number in parentheses is the 95% confidence interval.

The results provided above are summarized in Figure 5. The prehospitalization and preintubation periods of intubated non-survivors and ECMO patients appeared to be longer than those of intubated survivors (no direct comparison).

Finally, sensitivity analysis focusing on regional differences and patient backgrounds was performed. The regions where the studies were conducted were classified into three groups: Europe, the US, and East Asia. The intubation period was 14.87 days (10.99–18.76 days), 12.61 days (10.50–14.72 days), and 9.66 days (5.07–14.25 days) in Europe, the US, and East Asia (Supplementary Figure 3), and the intubation-death period was 13.05 days (9.53–16.58 days), 11.34 days (8.06–14.61 days), and 5.39 days (-1.14–11.91 days), respectively. Both the intubation period and intubation-death period tended to be longer in the US and Europe than in East Asia. Nevertheless, the mean age of intubated survivors did not differ much among Europe [64.85 (60.84–68.85)], the US [58.09 (49.32–66.87)], and Asia [61.64 (57.20–66.07)]; the mean age of intubated non-survivors was 67.86 (65.86–69.86) in Europe and 70.67 (61.88–79.46) in the US [one paper reported that the mean age of non-survivors in China was 65 (±4)]. We also analyzed age, sex, and comorbidities (diabetes mellitus and hypertension) but found no significant differences (data not shown).

## Discussion

This study demonstrated the clinical course and differences among the clinical courses of intubated survivors, non-survivors, and ECMO patients with COVID-19. In this meta-analysis, intubation, intubation-death, and ECMO periods were 12.07 days (9.80–14.33 days), 10.14 days (8.18–12.10 days), and 14.72 days (10.57–18.87 days), respectively. The prehospitalization periods of intubated survivors, non-survivors, and ECMO patients were 6.15 days (4.61–7.69 days), 6.45 days (4.55–8.34 days), and 7.15 days (6.48–7.81 days), respectively, and the preintubation periods were 8.58 days (7.36–9.80 days), 9.14 days (7.26–11.01 days), and 10.54 days (9.18–11.90 days), respectively. According to sensitivity analysis, the intubation period in survivors and the intubation-death period were longer in the US and Europe than in East Asia.

For COVID-19, the intubation period in survivors and the intubation-death period appear to be more prolonged than those in patients intubated for other diseases or reasons. In addition, the intubation periods in survivors and non-survivors were 12.1 days and 10 days, respectively. In contrast, previously reported intubation periods in ICU patients, including postoperative patients, chronic obstructive pulmonary disease (COPD) patients, pneumocystis pneumonia survivors, acute respiratory distress syndrome (ARDS) patients, community-acquired pneumonia patients, and SARS-CoV-1 pneumonia survivors, were 3.3 ± 3 days (mortality 24.3%) (119), 6.8 ± 4.9 days (mortality 13%) (120), 7.7 ± 8.2 days (121), 8.8 (± 6) days (122), 10–11 days (123), 12.1 ± 6.1 days (124), and 7.3–15 days (mean days are calculated from each original datum) (125–127), respectively. One study comparing COVID-19 with influenza found that the intubation period in COVID-19 patients was longer than that in influenza patients (16.2 vs. 7.3 days) (127). Thus, the intubation period in COVID-19 survivors is prolonged compared with that in patients intubated due to COPD, HIV-PCP, and influenza. However, approximately the same duration has been observed for patients intubated due to community-acquired pneumonia and SARS-CoV-1 or COVID-19.

Moreover, the ECMO period in COVID-19 patients was not longer than that in patients with ARDS for other reasons. Although ECMO is commonly used during cardiac surgery, severe ARDS patients (aPaO_2_:FiO_2_ of <80 mmHg, a Murray score >3.0 and pH <7.20) have been treated with ECMO, improving the survival rate to more than 50% (128, 129). Accordingly, critical COVID-19 patients also receive ECMO. In our meta-analysis, the ECMO period with COVID-19 was 14.72 days (10.57–18.87 days); in previous reports, the ECMO period in severe ARDS patients was 10.3 ± 7.5 (mean days were calculated from the data) days (128) and 15 ± 13 days (129), and that in severe ARDS patients with influenza was 10 days (130). These data indicate that the ECMO period in COVID-19 patients is not as long as we expected. We presume that time is needed to improve both ARDS caused by COVID-19 and ARDS caused by other reasons, as recovery in patients with critical ARDS is difficult.

In this study, the preintubation period was longer in intubated survivors than in intubated non-survivors or ECMO patients. This tendency was also observed when assessing data for the prehospitalization and hospitalization-intubation periods, despite no direct comparison. Indeed, the time from symptom onset to pneumonia was longer in COVID-19 patients with severe disease than in those without severe disease (131), the time from symptom onset to dyspnea and hospitalization in ICU COVID-19 patients was longer than that in non-ICU COVID-19 patients, and the time from symptom onset to ICU admission tended to be longer in COVID-19 non-survivors than in COVID-19 survivors (10).

There are two possibilities for these findings. First, hospitalization delay and lack of medical resources may contribute to the result. COVID-19 pandemic forces people to self-restraint, and it leads to hospitalization delay. Moreover, a shortage of ventilators also leads to intubation delay and poor prognosis (132–134). In fact, the hospitalization-intubation period among non-survivors, and ECMO patients tended to be longer than that among intubated survivors; the hospitalization-intubation period among intubated survivors, non-survivors, and ECMO patients with COVID-19 was 2.62 days (1.66–3.58 days), 3.28 days (2.15–4.41 days), and 3.39 (2.08–4.69 days), respectively. Second, a critical condition itself leads to a long prehospitalization period. Although the mechanism has yet to be elucidated, some of the COVID-19 patients experience asymptomatic hypoxia, which is also called “silent hypoxia.” In COVID-19 patients, 28.1% of hospitalized patients are estimated to have “silent hypoxia;” 33% of hospitalized patients with “silent hypoxia” are admitted to the ICU, and 25.9% of hospitalized patients with “silent hypoxia” die (135). Hence, “silent hypoxia” itself is a critical condition that leads to a long prehospitalization period. In this situation, monitoring blood oxygen, early hospitalization with oxygen supplementation, and systemic management arguably lead to a better prognosis.

The reasons why the intubation period was shorter in East Asia than in the US and Europe may include selection bias, information bias, differences in treatment, ventilator and ICU bed availability, race, and genetics. We detected bias concerning the East Asia data, which showed minor variations in regions and faculties compared to those from the US and Europe because East Asia's data were mainly from China, especially Wuhan. The shortage of ventilators and ICU beds has been highlighted in the US and Europe (132, 136), and it arguably contributed to a delay of intubation and a prolonged clinical course. Race and genetic background are also possible reasons for the observed clinical differences among regions. For example, data from the US show that Asians were hospitalized less but that Black and Hispanics were hospitalized more (137, 138). Sixteen percent of the genes were derived from Neanderthals, one of the prognostic factors maintained in Europe (almost 0% in East Asia) (139). GWASs have revealed that SNPs and blood type, the percentages of which differ among races and regions, are also prognostic factors. Thus, an international cohort study is needed to reveal the difference in clinical course between race and region.

## Limitations

There were also some limitations in this meta-analysis. Although many studies were included, more studies and patients are needed. Furthermore, heterogeneity was high because it was difficult to standardize the patients' backgrounds. This study revealed the clinical course of survivors and non-survivors, but a direct comparison with survivors and non-survivors is still needed. Clinical information, for example, age, BMI, cardiac disease, kidney disease, and chronic obstructive disease, was not sufficiently described in the cases we included, and articles in some of the countries reporting were limited. Social information was also not described in the cases we included; however, whether social information, such as patient or national income level, affects the clinical course is of great interest. Moreover, differences in viral strain and treatment including anti-inflammatory treatment, because of the study period. In general, comparing clinical data with our data will reveal more knowledge about COVID-19.

## Conclusions

Our data indicate that prehospitalization and intubation periods were longer in intubated non-survivors and ECMO patients than in intubated survivors with COVID-19. These periods might serve as predictors of disease severity or death and support therapeutic strategy determination. In the near future, viral strains and treatments should be taken into account when evaluating the clinical course of COVID-19.

## Data Availability Statement

The original contributions presented in the study are included in the article/Supplementary Material, further inquiries can be directed to the corresponding author.

## Author Contributions

KF and TM designed the study, carried out the literature search, independently acquired the data, screened the records, extracted the data, assessed the risk of bias, and performed the statistical analyses. All authors contributed to the article and approved the submitted version.

## Funding

This work was supported by research grants from the Center of Innovation program (COISTREAM) from the Ministry of Education, Culture, Sports, Science and Technology of Japan (MEXT) (to AK), the Japan Society for the Promotion of Science (JSPS) KAKENHI (JP18H05282 to AK), the Japan Agency for Medical Research and Development (AMED) (J200705023, J200705710, J200705049, JP18cm016335, JP18cm059042, and J210705582 to AK), a grant from the Kansai Economic Federation (KANKEIREN), and Grants from Mitsubishi Foundation (to AK). This work was also supported by the Japan Society for the Promotion of Science (JSPS) KAKENHI (JP21K16287 to TM).

## Conflict of Interest

The authors declare that the research was conducted in the absence of any commercial or financial relationships that could be construed as a potential conflict of interest.

## Publisher's Note

All claims expressed in this article are solely those of the authors and do not necessarily represent those of their affiliated organizations, or those of the publisher, the editors and the reviewers. Any product that may be evaluated in this article, or claim that may be made by its manufacturer, is not guaranteed or endorsed by the publisher.

## Abbreviations

CIs: confidence intervals
COVID-19: Coronavirus disease 2019
ECMO: extracorporeal membrane oxygenation
ECMO-death period: duration from ECMO initiation to death
ECMO period: duration from ECMO initiation to decannulation
hospitalization-death period: duration from hospitalization to death
hospitalization-discharge period: duration from hospitalization to discharge
hospitalization-ECMO period: duration from hospitalization to ECMO initiation
hospitalization-intubation period: duration from hospitalization to intubation
ICU: intensive care unit
intubation-death period: duration from intubation to death
intubation-ECMO period: duration from intubation to ECMO initiation
intubation period: duration from intubation to extubation
IQR: interquartile range
preECMO period: duration from symptom onset to ECMO initiation
prehospitalization period: duration from symptom onset to hospitalization
preintubation period: duration from symptom onset to intubation
SARS-CoV-1: severe acute respiratory syndrome coronavirus 1
SARS-CoV-2: severe acute respiratory syndrome coronavirus 2
SD: standard deviation
symptom-death period: duration from symptom onset to death.
