# Supplementary material for: Longer Prehospitalization and Preintubation Periods in Intubated Non-survivors and ECMO Patients With COVID-19: A Systematic Review and Meta-Analysis
Source: Front Med (Lausanne). 2021 Oct 15;8:727101. doi: 10.3389/fmed.2021.727101 (PMC8554002; doi:10.3389/fmed.2021.727101)
Supplement: Supplementary file 1 [file Data_Sheet_1.PDF]

# Longer prehospitalization and preintubation periods in intubated nonsurvivors and ECMO patients with COVID-19: A systematic review and meta-analysis

Kenji Funakoshi,[1] Takayoshi Morita,[1] Atsushi Kumanogoh.[1,2,3]

[1]Department of Respiratory Medicine and Clinical Immunology, Osaka University Graduate School of Medicine, Suita, Osaka, Japan.

[2]Department of Immunopathology, Immunology Frontier Research Center (WPI-IFReC), Osaka University, Suita, Osaka, Japan.

[3]Integrated Frontier Research for Medical Science Division, Institute for Open and Transdisciplinary Research Initiatives (OTRI), Osaka University, Suita, Osaka, Japan

***Corresponding author**

Takayoshi Morita, Department of Respiratory Medicine and Clinical Immunology, Graduate School of Medicine, Osaka University, Suita, Osaka, Japan Address: 2-2, Yamadaoka, Suita, Osaka 565-0871, Japan E-mail: t-morita@imed3.med.osaka-u.ac.jp

**Supplement**

**Table S1.** PRISMA checklist.

| Section/topic | # | Checklist item | Reported on page # |
|---|---|---|---|
| **TITLE** | | | |
| Title | 1 | Identify the report as a systematic review, meta-analysis, or both. | 1 |
| **ABSTRACT** | | | |
| Structured summary | 2 | Provide a structured summary including as applicable: background; objectives; data sources; study eligibility criteria, participants, and interventions; study appraisal and synthesis methods; results; limitations; conclusions and implications of key findings; systematic review registration number. | # 3 |
| **INTRODUCTION** | | | |
| Rationale | 3 | Describe the rationale for the review in the context of what is already known. | # 4-5 |
| Objectives | 4 | Provide an explicit statement of questions being addressed with reference to participants, interventions, comparisons, outcomes, and study design (PICOS). | # 4-5 |
| **METHODS** | | | |
| Protocol and registration | 5 | Indicate if a review protocol exists, if and where it can be accessed (e.g., Web address), and, if available, provide registration information including registration number. | # 5-7 |

| | | | |
|---|---|---|---|
| Eligibility criteria | 6 | Specify study characteristics (e.g., PICOS, length of follow-up) and report characteristics (e.g., years considered, language, publication status) used as criteria for eligibility, giving rationale. | # 5-6 |
| Information sources | 7 | Describe all information sources (e.g., databases with dates of coverage, contact with study authors to identify additional studies) in the search and date last searched. | # 5-6 |
| Search | 8 | Present full electronic search strategy for at least one database, including any limits used, such that it could be repeated. | # 5-6 |
| Study selection | 9 | State the process for selecting studies (i.e., screening, eligibility, included in systematic review, and, if applicable, included in the meta-analysis). | # 5-6 |
| Data collection process | 10 | Describe method of data extraction from reports (e.g., piloted forms, independently, in duplicate) and any processes for obtaining and confirming data from investigators. | # 6-7 |
| Data items | 11 | List and define all variables for which data were sought (e.g., PICOS, funding sources) and any assumptions and simplifications made. | # 6-7 |
| Risk of bias in individual studies | 12 | Describe methods used for assessing risk of bias of individual studies (including specification of whether this was done at the study or outcome level), and how this information is to be used in any data synthesis. | # 6-7 |
| Summary measures | 13 | State the principal summary measures (e.g., risk ratio, difference in means). | # 7 |
| Synthesis of results | 14 | Describe the methods of handling data and combining results of studies, if done, including measures of | # 6-7 |

| | | | |
|---|---|---|---|
| | | consistency (e.g., $I^2$) for each meta-analysis. | |
| Risk of bias across studies | 15 | Specify any assessment of risk of bias that may affect the cumulative evidence (e.g., publication bias, selective reporting within studies). | # 6-7 |
| Additional analyses | 16 | Describe methods of additional analyses (e.g., sensitivity or subgroup analyses, meta-regression), if done, indicating which were prespecified. | # 7 |
| **RESULTS** | | | |
| Study selection | 17 | Give numbers of studies screened, assessed for eligibility, and included in the review, with reasons for exclusions at each stage, ideally with a flow diagram. | # 7-8, Fig 1 |
| Study characteristics | 18 | For each study, present characteristics for which data were extracted (e.g., study size, PICOS, follow-up period) and provide the citations. | Table |
| Risk of bias within studies | 19 | Present data on risk of bias of each study and, if available, any outcome level assessment (see item 12). | Table S2<br>Fig S1 |
| Results of individual studies | 20 | For all outcomes considered (benefits or harms), present, for each study: (a) simple summary data for each intervention group (b) effect estimates and confidence intervals, ideally with a forest plot. | # 8-10<br>Fig 2-5<br>Fig S2-3 |
| Synthesis of results | 21 | Present results of each meta-analysis done, including confidence intervals and measures of consistency. | # 11-13<br>Fig 2-5 |

| | | | |
|---|---|---|---|
| | | | Fig S2-3 |
| Risk of bias across studies | 22 | Present results of any assessment of risk of bias across studies (see Item 15). | Table S3 |
| Additional analysis | 23 | Give results of additional analyses, if done (e.g., sensitivity or subgroup analyses, meta-regression [see Item 16]). | Fig S3 |
| **DISCUSSION** | | | |
| Summary of evidence | 24 | Summarize the main findings including the strength of evidence for each main outcome; consider their relevance to key groups (e.g., healthcare providers, users, and policy makers). | # 10 |
| Limitations | 25 | Discuss limitations at study and outcome level (e.g., risk of bias), and at review-level (e.g., incomplete retrieval of identified research, reporting bias). | # 13 |
| Conclusions | 26 | Provide a general interpretation of the results in the context of other evidence, and implications for future research. | # 14 |
| **FUNDING** | | | |
| Funding | 27 | Describe sources of funding for the systematic review and other support (e.g., supply of data); role of funders for the systematic review. | #14-15 |

**Table S2.** Study Quality Assessment Tools (Quality Assessment of Systematic Reviews and Meta-Analyses) from the National Heart, Lung, and Blood Institute (NHLBI)

| IMV/ECMO | Study | Study design | Criteria | | | | | | | | | |
|---|---|---|---|---|---|---|---|---|---|---|---|---|
| | | | 1[a] | 2[b] | 3[c] | 4[d] | 5[e] | 6[f] | 7[g] | 8[h] | 9[i] | Total points |
| IMV | Abe et al. | Case series | Yes | Yes | NR | NA | No | Yes | Yes | NA | Yes | 5 |
| IMV | Argenziano et al. | Case series | Yes | Yes | Yes | Yes | No | Yes | Yes | Yes | Yes | 8 |
| IMV | Barrasa et al. | Case series | Yes | Yes | Yes | Yes | No | Yes | Yes | Yes | Yes | 8 |
| IMV | Beigmohammadi et al. | Case series | Yes | Yes | NR | NA | No | Yes | Yes | NA | Yes | 5 |
| IMV | Bhatraju et al. | Case series | Yes | Yes | NR | NA | No | Yes | Yes | Yes | Yes | 6 |
| IMV | Cauchois et al. | Case series | Yes | Yes | NR | No | Yes | Yes | Yes | Yes | Yes | 7 |
| IMV | Chen et al. | Case series | Yes | Yes | Yes | NA | No | Yes | Yes | NA | Yes | 6 |
| IMV | Christie 3rd et al. | Case series | Yes | Yes | NR | No | Yes | Yes | Yes | NA | Yes | 6 |
| IMV | Cummings et al. | Case control | Yes | Yes | Yes | NA | No | Yes | Yes | Yes | Yes | 7 |
| IMV | Dai et al. | Case control | Yes | Yes | NR | Yes | Yes | Yes | Yes | Yes | Yes | 8 |
| IMV | Dastan et al. | Case control | Yes | Yes | Yes | Yes | Yes | Yes | Yes | Yes | Yes | 9 |

| | | | | | | | | | | | | |
|---|---|---|---|---|---|---|---|---|---|---|---|---|
| IMV | De Luca et al. | Case series | Yes | Yes | Yes | Yes | Yes | Yes | Yes | Yes | Yes | 9 |
| IMV | Dogan et al. | Case series | Yes | Yes | NR | No | Yes | Yes | Yes | NA | Yes | 6 |
| IMV | Elder et al. | Case series | Yes | Yes | NR | NA | No | Yes | Yes | NA | Yes | 5 |
| IMV | Falces-Romero et al. | Case series | Yes | Yes | NR | NA | No | Yes | Yes | NA | Yes | 5 |
| IMV | Flikweert et al. | Case series | Yes | Yes | NR | NA | No | Yes | Yes | NA | Yes | 5 |
| IMV | Gavin et al. | Case series | Yes | Yes | Yes | Yes | No | Yes | Yes | Yes | Yes | 8 |
| IMV | Grasselli et al. | Case control | Yes | Yes | Yes | Yes | No | Yes | Yes | Yes | Yes | 8 |
| IMV | Grein et al. | Case control | Yes | Yes | NR | No | Yes | Yes | Yes | Yes | Yes | 7 |
| IMV | Halvatsiotis et al. | Case control | Yes | Yes | Yes | Yes | No | Yes | Yes | Yes | Yes | 8 |
| IMV | Hernandez-Romieu et al. | Case control | Yes | Yes | NR | Yes | No | Yes | Yes | Yes | Yes | 7 |
| IMV | Kato et al. | Case series | Yes | Yes | NR | Yes | No | Yes | Yes | Yes | Yes | 7 |
| IMV | Ketcham et al. | Case series | Yes | Yes | NR | Yes | No | Yes | Yes | Yes | Yes | 7 |
| IMV | Kewan et al. | Case series | Yes | Yes | Yes | No | Yes | Yes | Yes | NA | Yes | 7 |
| IMV | Khullar et al. | Case control | Yes | Yes | Yes | Yes | No | Yes | Yes | Yes | Yes | 8 |
| IMV | Konopka et al. | Case series | Yes | Yes | NR | Yes | No | Yes | Yes | Yes | Yes | 7 |
| IMV | Krishnan et al. | Case control | Yes | Yes | Yes | Yes | No | Yes | Yes | Yes | Yes | 8 |

| | | | | | | | | | | | | | |
|---|---|---|---|---|---|---|---|---|---|---|---|---|---|
| IMV | Kristinsson et al. | Case series | Yes | Yes | Yes | NA | No | Yes | Yes | Yes | Yes | 7 |
| IMV | Lê et al. | Case series | Yes | Yes | No | NA | No | Yes | Yes | Yes | Yes | 6 |
| IMV | LeBrun et al. | Case series | Yes | Yes | NR | Yes | No | Yes | Yes | Yes | Yes | 7 |
| IMV | Lechien et al. | Case series | Yes | Yes | Yes | Yes | No | Yes | Yes | Yes | Yes | 8 |
| IMV | Lee et al. | Case series | Yes | Yes | Yes | NA | No | Yes | Yes | Yes | Yes | 7 |
| IMV | Liu et al. | Case control | Yes | Yes | NR | Yes | Yes | Yes | Yes | Yes | Yes | 8 |
| IMV | Lowe et al. | Case series | Yes | Yes | Yes | NA | No | Yes | Yes | NA | Yes | 6 |
| IMV | Maritati et al. | Case series | Yes | Yes | NR | NA | No | Yes | Yes | NA | Yes | 5 |
| IMV | Morassi et al. | Case series | Yes | Yes | NR | NA | No | Yes | Yes | NA | Yes | 5 |
| IMV | Morillas et al. | Case series | Yes | Yes | NR | No | Yes | Yes | Yes | Yes | Yes | 7 |
| IMV | Navarro-Millán et al. | Case series | Yes | Yes | NR | NA | Yes | Yes | Yes | NA | Yes | 6 |
| IMV | Novelli et al. | Case control | Yes | Yes | Yes | Yes | No | Yes | Yes | Yes | Yes | 8 |
| IMV | Pan et al. | Case series | Yes | Yes | NR | No | Yes | Yes | Yes | NA | Yes | 6 |
| IMV | Peng et al. | Case series | Yes | Yes | NR | No | Yes | Yes | Yes | NA | Yes | 6 |
| IMV | Plotnikow et al. | Case control | Yes | Yes | Yes | Yes | No | Yes | Yes | Yes | Yes | 8 |
| IMV | Radnis et al. | Case series | Yes | Yes | NR | NA | No | Yes | Yes | NA | Yes | 5 |

| | | | | | | | | | | | | | |
|---|---|---|---|---|---|---|---|---|---|---|---|---|---|
| IMV | Riker et al. | Case series | Yes | Yes | NR | NA | No | Yes | Yes | NA | Yes | 5 |
| IMV | Rizo-Téllez et al. | Case control | Yes | Yes | NR | Yes | No | Yes | Yes | Yes | Yes | 7 |
| IMV | Sakr et al. | Case series | Yes | Yes | NR | No | No | Yes | Yes | NA | Yes | 5 |
| IMV | Schaefer et al. | Case series | Yes | Yes | NR | NA | No | Yes | Yes | NA | Yes | 5 |
| IMV | Shen et al. | Case series | Yes | Yes | NR | No | Yes | Yes | Yes | Yes | Yes | 7 |
| IMV | Singh et al. | Case series | Yes | Yes | NR | NA | No | Yes | Yes | Yes | Yes | 6 |
| IMV | So et al. | Case series | Yes | Yes | NR | No | Yes | Yes | Yes | NA | Yes | 6 |
| IMV | Søvik et al. | Case control | Yes | Yes | NR | Yes | No | Yes | Yes | Yes | Yes | 7 |
| IMV | Stony Brook COVID-19 Research Consortium | Case control | Yes | Yes | NR | Yes | No | Yes | Yes | Yes | Yes | 7 |
| IMV | Wali et al. | Case series | Yes | Yes | NR | No | Yes | Yes | Yes | NA | Yes | 6 |
| IMV | Wang et al. | Case control | Yes | Yes | NR | Yes | No | Yes | Yes | Yes | Yes | 7 |
| IMV | Wang et al. | Case series | Yes | Yes | NR | NA | No | Yes | Yes | NA | Yes | 5 |
| IMV | Weiskopf et al. | Case series | Yes | Yes | NR | NA | No | Yes | Yes | Yes | Yes | 6 |
| IMV | Wilk et al. | Case series | Yes | Yes | Yes | NA | No | Yes | Yes | Yes | Yes | 7 |
| IMV | Zhang et al. | Case series | Yes | Yes | Yes | Yes | No | Yes | Yes | Yes | Yes | 8 |
| IMV | Ziehr et al. | Case series | Yes | Yes | Yes | NA | No | Yes | Yes | Yes | Yes | 7 |

| | | | | | | | | | | | | |
|---|---|---|---|---|---|---|---|---|---|---|---|---|
| ECMO | Akhtar et al. | Case series | Yes | Yes | Yes | NA | No | Yes | Yes | Yes | Yes | 7 |
| ECMO | Alnababteh et al. | Case control | Yes | Yes | Yes | Yes | No | Yes | Yes | Yes | Yes | 8 |
| ECMO | Beyls et al. | Case series | Yes | Yes | NR | NA | No | Yes | Yes | NA | Yes | 5 |
| ECMO | Charlton et al. | Case series | Yes | Yes | Yes | NA | No | Yes | Yes | Yes | Yes | 7 |
| ECMO | Dastan et al. | Case control | Yes | Yes | Yes | Yes | Yes | Yes | Yes | Yes | Yes | 9 |
| ECMO | Falcoz et al. | Case series | Yes | Yes | Yes | NA | No | Yes | Yes | Yes | Yes | 7 |
| ECMO | Goursaud et al. | Case series | Yes | Yes | NR | NA | No | Yes | Yes | NA | Yes | 5 |
| ECMO | Grein et al. | Case control | Yes | Yes | NR | No | Yes | Yes | Yes | Yes | Yes | 7 |
| ECMO | Guihaire et al. | Case series | Yes | Yes | NR | NA | No | Yes | Yes | Yes | Yes | 6 |
| ECMO | Guo et al. | Case series | Yes | Yes | NR | NA | No | Yes | Yes | NA | Yes | 5 |
| ECMO | Heman-Ackah et al. | Case series | Yes | Yes | NR | NA | No | Yes | Yes | NA | Yes | 5 |
| ECMO | Huette et al. | Case series | Yes | Yes | Yes | NA | No | Yes | Yes | NA | Yes | 6 |
| ECMO | Jäckel et al. | Case series | Yes | Yes | Yes | Yes | No | Yes | Yes | Yes | Yes | 8 |
| ECMO | Jacobs et al. | Case control | Yes | Yes | Yes | Yes | No | Yes | Yes | Yes | Yes | 8 |
| ECMO | Kon et al. | Case series | Yes | Yes | Yes | NA | No | Yes | Yes | Yes | Yes | 7 |
| ECMO | Le Breton et al. | Case series | Yes | Yes | Yes | NA | No | Yes | Yes | NA | Yes | 6 |

| | | | | | | | | | | | | |
|---|---|---|---|---|---|---|---|---|---|---|---|---|
| ECMO | Li et al. | Case series | Yes | Yes | Yes | NA | No | Yes | Yes | NA | Yes | 6 |
| ECMO | Liu et al. | Case control | Yes | Yes | NR | Yes | Yes | Yes | Yes | Yes | Yes | 8 |
| ECMO | Liu et al. | Case control | Yes | Yes | Yes | Yes | No | Yes | Yes | Yes | Yes | 8 |
| ECMO | Loforte et al. | Case series | Yes | Yes | Yes | NA | No | Yes | Yes | NA | Yes | 6 |
| ECMO | Matsunaga et al. | Case series | Yes | Yes | Yes | NA | No | Yes | Yes | Yes | Yes | 7 |
| ECMO | Miike et al. | Case series | Yes | Yes | NR | NA | No | Yes | Yes | Yes | Yes | 6 |
| ECMO | Mustafa et al. | Case series | Yes | Yes | Yes | NA | No | Yes | Yes | NA | Yes | 6 |
| ECMO | Osho et al. | Case series | Yes | Yes | Yes | NA | No | Yes | Yes | NA | Yes | 6 |
| ECMO | Ronit et al. | Case series | Yes | Yes | No | NA | No | Yes | Yes | NA | Yes | 5 |
| ECMO | Schmidt et al. | Case control | Yes | Yes | Yes | Yes | No | Yes | Yes | Yes | Yes | 8 |
| ECMO | Shih et al. | Case series | Yes | Yes | Yes | NA | No | Yes | Yes | Yes | Yes | 7 |
| ECMO | Sultan et al. | Case control | Yes | Yes | Yes | NA | No | Yes | Yes | NA | Yes | 6 |
| ECMO | Usman et al. | Case control | Yes | Yes | NR | Yes | No | Yes | Yes | Yes | Yes | 7 |
| ECMO | Xu et al. | Case series | Yes | Yes | Yes | NA | No | Yes | Yes | Yes | Yes | 7 |
| ECMO | Xuan et al. | Case series | Yes | Yes | No | NA | No | Yes | Yes | NA | Yes | 5 |
| ECMO | Yang et al. | Case control | Yes | Yes | Yes | Yes | No | Yes | Yes | Yes | Yes | 8 |

| | | | | | | | | | | | | | |
|---|---|---|---|---|---|---|---|---|---|---|---|---|---|
| ECMO | Zayat et al. | Case control | Yes | Yes | Yes | Yes | No | Yes | Yes | Yes | Yes | 8 |
| ECMO | Zeng et al. | Case series | Yes | Yes | NR | NA | No | Yes | Yes | Yes | Yes | 6 |
| ECMO | Zeng et al. | Case series | Yes | Yes | Yes | Yes | No | Yes | Yes | Yes | Yes | 8 |
| ECMO | Zhang et al. | Case control | Yes | Yes | Yes | Yes | No | Yes | Yes | Yes | Yes | 8 |
| ECMO | Zhang et al. | Case series | Yes | Yes | NR | Yes | No | Yes | Yes | Yes | Yes | 7 |
| ECMO | Zheng et al. | Case control | Yes | Yes | Yes | Yes | No | Yes | Yes | Yes | Yes | 7 |

[*]The total score was calculated based on the study quality assessment tools from the NHLBI.

NA, not applicable; NR, not reported

[a]Criterion 1: Was the study question or objective clearly stated?

[b]Criterion 2: Was the study population clearly and fully described, including a case definition?

[c]Criterion 3: Were the cases consecutive?

[d]Criterion 4: Were the subjects comparable?

[e]Criterion 5: Was the intervention clearly described?

[f]Criterion 6: Were the outcome measures clearly defined, valid, reliable, and implemented consistently across all study participants?

[g]Criterion 7: Was the length of follow-up adequate?

[h]Criterion 8: Were the statistical methods well-described?

[i]Criterion 9: Were the results well-described?

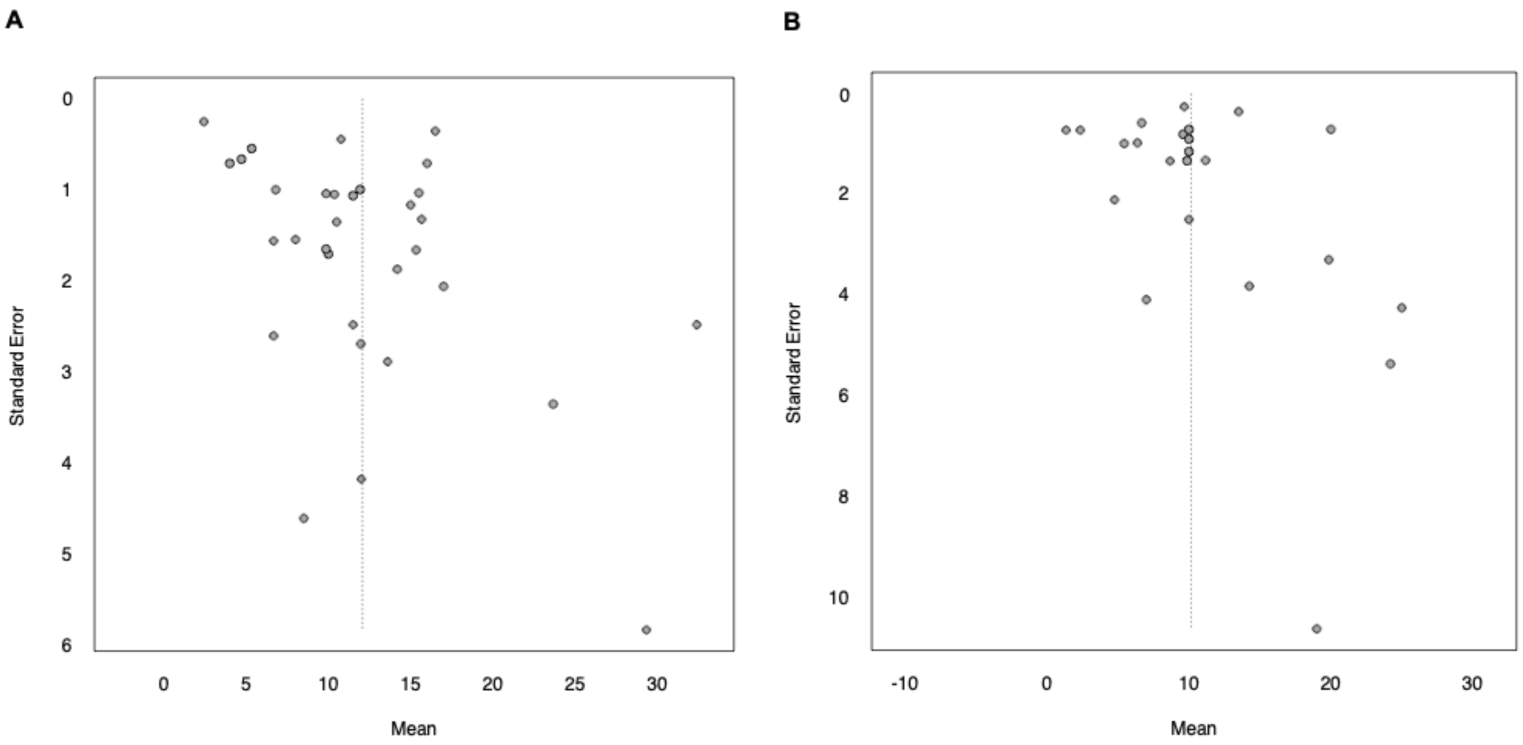


**Figure S1. Funnel plot for the intubation period of intubated COVID-19 survivors (A) and the intubation-death period of intubated COVID-19 nonsurvivors (B).**

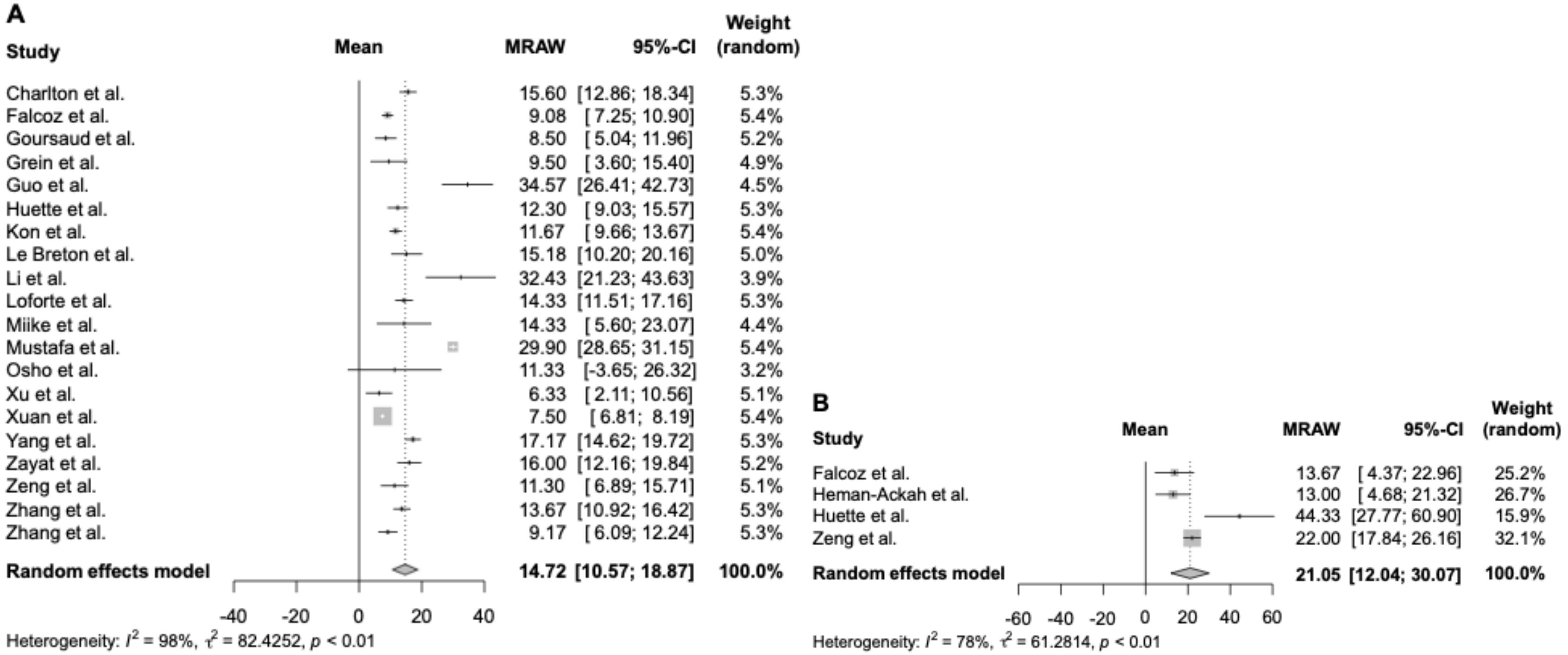


**Figure S2. A meta-analysis of the ECMO period (A) and the ECMO-death period (B) was calculated using the random effects model.** MRAW: the raw data of mean. 95% CI: 95% confidence interval.

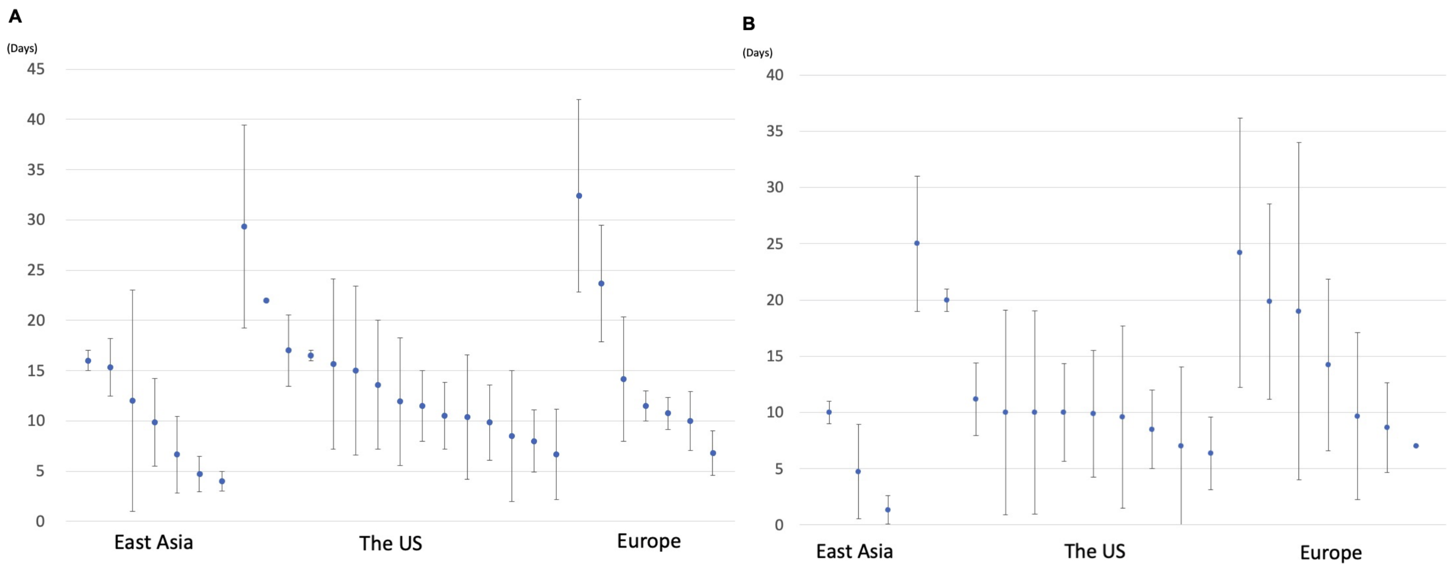


**Figure S3. Sensitivity analysis based on regional differences.** The mean days and standard deviation (SD) of the intubation period in intubated survivors (**A**) and the intubation-death period in intubated nonsurvivors (**B**) of each study were plotted.

**Reference**(1–94)


1. Abe T, Izumo T, Ueda A, Hayashi M, Ishibashi Y. Successful treatment of two Japanese ESRD cases with severe COVID-19 pneumonia. *CEN Case Rep* (2021) **10**:42–45. doi:10.1007/s13730-020-00512-7

2. Argenziano MG, Bruce SL, Slater CL, Tiao JR, Baldwin MR, Barr RG, Chang BP, Chau KH, Choi JJ, Gavin N, et al. Characterization and clinical course of 1000 patients with coronavirus disease 2019 in New York: retrospective case series. *BMJ* (2020) **369**:m1996. doi:10.1136/bmj.m1996

3. Barrasa H, Rello J, Tejada S, Martín A, Balziskueta G, Vinuesa C, Fernández-Miret B, Villagra A, Vallejo A, San Sebastián A, et al. SARS-CoV-2 in Spanish Intensive Care Units: Early experience with 15-day survival in Vitoria. *Anaesth Crit Care Pain Med* (2020) **39**:553–561. doi:10.1016/j.accpm.2020.04.001

4. Beigmohammadi MT, Jahanbin B, Safaei M, Amoozadeh L, Khoshavi M, Mehrtash V, Jafarzadeh B, Abdollahi A. Pathological Findings of Postmortem Biopsies From Lung, Heart, and Liver of 7 Deceased COVID-19 Patients. *Int J Surg Pathol* (2021) **29**:135–145. doi:10.1177/1066896920935195

5. Bhatraju PK, Ghassemieh BJ, Nichols M, Kim R, Jerome KR, Nalla AK, Greninger AL, Pipavath S, Wurfel MM, Evans L, et al. Covid-19 in Critically Ill Patients in the Seattle Region - Case Series. *N Engl J Med* (2020) **382**:2012–2022. doi:10.1056/NEJMoa2004500

6. Cauchois R, Koubi M, Delarbre D, Manet C, Carvelli J, Blasco VB, Jean R, Fouche L, Bornet C, Pauly V, et al. Early IL-1 receptor blockade in severe inflammatory respiratory failure complicating COVID-19. *Proc Natl Acad Sci U S A* (2020) **117**:18951–18953. doi:10.1073/pnas.2009017117

7. Chen N, Zhou M, Dong X, Qu J, Gong F, Han Y, Qiu Y, Wang J, Liu Y, Wei Y, et al. Epidemiological and clinical characteristics of 99 cases of 2019 novel coronavirus pneumonia in Wuhan, China: a descriptive study. *Lancet* (2020) **395**:507–513. doi:10.1016/S0140-6736(20)30211-7

8. Christie DB 3rd, Nemec HM, Scott AM, Buchanan JT, Franklin CM, Ahmed A, Khan MS, Callender CW, James EA, Christie AB, et al. Early outcomes with utilization of tissue plasminogen activator in COVID-19-associated respiratory distress: A series of five cases. *J Trauma Acute Care Surg* (2020) **89**:448–452. doi:10.1097/TA.0000000000002787

9. Cummings MJ, Baldwin MR, Abrams D, Jacobson SD, Meyer BJ, Balough EM, Aaron JG, Claassen J, Rabbani LE, Hastie J, et al. Epidemiology, clinical course, and outcomes of critically ill adults with COVID-19 in New York City: a prospective cohort study. *Lancet* (2020) **395**:1763–1770. doi:10.1016/S0140-6736(20)31189-2

10. Dai M, Liu D, Liu M, Zhou F, Li G, Chen Z, Zhang Z, You H, Wu M, Zheng Q, et al. Patients with Cancer Appear More Vulnerable to SARS-CoV-2: A Multicenter Study during the COVID-19 Outbreak. *Cancer Discov* (2020) **10**:783–791. doi:10.1158/2159-

8290.CD-20-0422

11. Dastan F, Saffaei A, Haseli S, Marjani M, Moniri A, Abtahian Z, Abedini A, Kiani A, Seifi S, Jammati H, et al. Promising effects of tocilizumab in COVID-19: A non-controlled, prospective clinical trial. *Int Immunopharmacol* (2020) **88**:106869. doi:10.1016/j.intimp.2020.106869

12. De Luca G, Cavalli G, Campochiaro C, Della-Torre E, Angelillo P, Tomelleri A, Boffini N, Tentori S, Mette F, Farina N, et al. GM-CSF blockade with mavrilimumab in severe COVID-19 pneumonia and systemic hyperinflammation: a single-centre, prospective cohort study. *Lancet Rheumatol* (2020) **2**:e465–e473. doi:10.1016/S2665-9913(20)30170-3

13. Dogan L, Kaya D, Sarikaya T, Zengin R, Dincer A, Akinci IO, Afsar N. Plasmapheresis treatment in COVID-19-related autoimmune meningoencephalitis: Case series. *Brain Behav Immun* (2020) **87**:155–158. doi:10.1016/j.bbi.2020.05.022

14. Elder C, Bawa S, Anderson D, Atkinson S, Etzel J, Moritz T. Expectant management of pneumothorax in intubated COVID-19 positive patients: a case series. *J Cardiothorac Surg* (2020) **15**:263. doi:10.1186/s13019-020-01297-7

15. Falces-Romero I, Ruiz-Bastián M, Díaz-Pollán B, Maseda E, García-Rodríguez J, SARS-CoV-2 Working Group. Isolation of Aspergillus spp. in respiratory samples of patients with COVID-19 in a Spanish Tertiary Care Hospital. *Mycoses* (2020) doi:10.1111/myc.13155

16. Flikweert AW, Grootenboers MJJH, Yick DCY, du Mée AWF, van der Meer NJM, Rettig TCD, Kant MKM. Late histopathologic characteristics of critically ill COVID-19 patients: Different phenotypes without evidence of invasive aspergillosis, a case series. *J Crit Care* (2020) **59**:149–155. doi:10.1016/j.jcrc.2020.07.002

17. Gavin W, Campbell E, Zaidi S-A, Gavin N, Dbeibo L, Beeler C, Kuebler K, Abdel-Rahman A, Luetkemeyer M, Kara A. Clinical characteristics, outcomes and prognosticators in adult patients hospitalized with COVID-19. *Am J Infect Control* (2021) **49**:158–165. doi:10.1016/j.ajic.2020.07.005

18. Grasselli G, Greco M, Zanella A, Albano G, Antonelli M, Bellani G, Bonanomi E, Cabrini L, Carlesso E, Castelli G, et al. Risk Factors Associated With Mortality Among Patients With COVID-19 in Intensive Care Units in Lombardy, Italy. *JAMA Intern Med* (2020) **180**:1345–1355. doi:10.1001/jamainternmed.2020.3539

19. Grein J, Ohmagari N, Shin D, Diaz G, Asperges E, Castagna A, Feldt T, Green G, Green ML, Lescure F-X, et al. Compassionate Use of Remdesivir for Patients with Severe Covid-19. *N Engl J Med* (2020) **382**:2327–2336. doi:10.1056/NEJMoa2007016

20. Halvatsiotis P, Kotanidou A, Tzannis K, Jahaj E, Magira E, Theodorakopoulou M, Konstandopoulou G, Gkeka E, Pourzitaki C, Kapravelos N, et al. Demographic and clinical features of critically ill patients with COVID-19 in Greece: The burden of diabetes and obesity. *Diabetes Res Clin Pract* (2020) **166**:108331. doi:10.1016/j.diabres.2020.108331

21. Hernandez-Romieu AC, Adelman MW, Hockstein MA, Robichaux CJ, Edwards JA, Fazio JC, Blum JM, Jabaley CS, Caridi-Scheible M, Martin GS, et al. Timing of Intubation and Mortality Among Critically Ill Coronavirus Disease 2019 Patients: A Single-Center Cohort Study. *Crit Care Med* (2020) **48**:e1045–e1053. doi:10.1097/CCM.0000000000004600

22. Kato H, Shimizu H, Shibue Y, Hosoda T, Iwabuchi K, Nagamine K, Saito H, Sawada R, Oishi T, Tsukiji J, et al. Clinical course of 2019 novel coronavirus disease (COVID-19) in individuals present during the outbreak on the Diamond Princess cruise ship. *J Infect Chemother* (2020) **26**:865–869. doi:10.1016/j.jiac.2020.05.005

23. Ketcham SW, Adie SK, Malliett A, Abdul-Aziz AA, Bitar A, Grafton G, Konerman MC. Coronavirus Disease-2019 in Heart Transplant Recipients in Southeastern Michigan: A Case Series. *J Card Fail* (2020) **26**:457–461. doi:10.1016/j.cardfail.2020.05.008

24. Kewan T, Covut F, Al-Jaghbeer MJ, Rose L, Gopalakrishna KV, Akbik B. Tocilizumab for treatment of patients with severe COVID-19: A retrospective cohort study. *EClinicalMedicine* (2020) **24**:100418. doi:10.1016/j.eclinm.2020.100418

25. Khullar R, Shah S, Singh G, Bae J, Gattu R, Jain S, Green J, Anandarangam T, Cohen M, Madan N, et al. Effects of Prone Ventilation on Oxygenation, Inflammation, and Lung Infiltrates in COVID-19 Related Acute Respiratory Distress Syndrome: A Retrospective Cohort Study. *J Clin Med Res* (2020) **9**: doi:10.3390/jcm9124129

26. Konopka KE, Nguyen T, Jentzen JM, Rayes O, Schmidt CJ, Wilson AM, Farver CF, Myers JL. Diffuse alveolar damage (DAD) resulting from coronavirus disease 2019 Infection is Morphologically Indistinguishable from Other Causes of DAD. *Histopathology* (2020) **77**:570–578. doi:10.1111/his.14180

27. Krishnan S, Patel K, Desai R, Sule A, Paik P, Miller A, Barclay A, Cassella A, Lucaj J, Royster Y, et al. Clinical comorbidities, characteristics, and outcomes of mechanically ventilated patients in the State of Michigan with SARS-CoV-2 pneumonia. *J Clin Anesth* (2020) **67**:110005. doi:10.1016/j.jclinane.2020.110005

28. Kristinsson B, Kristinsdottir LB, Blondal AT, Thormar KM, Kristjansson M, Karason S, Sigvaldason K, Sigurdsson MI. Nationwide Incidence and Outcomes of Patients With Coronavirus Disease 2019 Requiring Intensive Care in Iceland. *Crit Care Med* (2020) **48**:e1102–e1105. doi:10.1097/CCM.0000000000004582

29. Lê MP, Jaquet P, Patrier J, Wicky P-H, Le Hingrat Q, Veyrier M, Kauv J, Sonneville R, Visseaux B, Laouénan C, et al. Pharmacokinetics of lopinavir/ritonavir oral solution to treat COVID-19 in mechanically ventilated ICU patients. *J Antimicrob Chemother* (2020) **75**:2657–2660. doi:10.1093/jac/dkaa261

30. LeBrun DG, Konnaris MA, Ghahramani GC, Premkumar A, DeFrancesco CJ, Gruskay JA, Dvorzhinskiy A, Sandhu MS, Goldwyn EM, Mendias CL, et al. Hip Fracture Outcomes During the COVID-19 Pandemic: Early Results From New York. *J Orthop Trauma* (2020) **34**:403–410. doi:10.1097/BOT.0000000000001849

31. Lechien JR, Chiesa-Estomba CM, De Siati DR, Horoi M, Le Bon SD, Rodriguez A, Dequanter D, Blecic S, El Afia F, Distinguin L, et al. Olfactory and gustatory dysfunctions as a clinical presentation of mild-to-moderate forms of the coronavirus disease (COVID-19): a multicenter European study. *Eur Arch Otorhinolaryngol* (2020) **277**:2251–2261. doi:10.1007/s00405-020-05965-1

32. Lee AJY, Chung CLH, Young BE, Ling LM, Ho BCH, Puah SH, Iqbal SR, Tan GP. Clinical course and physiotherapy intervention in 9 patients with COVID-19. *Physiotherapy* (2020) **109**:1–3. doi:10.1016/j.physio.2020.06.002

33. Liu J, Zhang S, Wu Z, Shang Y, Dong X, Li G, Zhang L, Chen Y, Ye X, Du H, et al. Clinical outcomes of COVID-19 in Wuhan, China: a large cohort study. *Ann Intensive Care* (2020) **10**:99. doi:10.1186/s13613-020-00706-3

34. Lowe A, Chang DD, Creek G. Multiple Fatalities in a Family Cluster of COVID-19 With Acute Respiratory Distress Syndrome. *Ochsner J* (2020) **20**:134–138. doi:10.31486/toj.20.0056

35. Maritati F, Cerutti E, Zuccatosta L, Fiorentini A, Finale C, Ficosecco M, Cristiano F, Capestro A, Balestra E, Taruscia D, et al. SARS-CoV-2 infection in kidney transplant recipients: Experience of the italian marche region. *Transpl Infect Dis* (2020) **22**:e13377. doi:10.1111/tid.13377

36. Morassi M, Bagatto D, Cobelli M, D'Agostini S, Gigli GL, Bnà C, Vogrig A. Stroke in patients with SARS-CoV-2 infection: case series. *J Neurol* (2020) **267**:2185–2192. doi:10.1007/s00415-020-09885-2

37. Morillas JA, Marco Canosa F, Srinivas P, Asadi T, Calabrese C, Rajendram P, Budev M, Poggio ED, Narayanan Menon KV, Gastman B, et al. Tocilizumab therapy in 5 solid and composite tissue transplant recipients with early ARDS due to SARS-CoV-2. *Am J Transplant* (2020) **20**:3191–3197. doi:10.1111/ajt.16080

38. Navarro-Millán I, Sattui SE, Lakhanpal A, Zisa D, Siegel CH, Crow MK. Use of Anakinra to Prevent Mechanical Ventilation in Severe COVID-19: A Case Series. *Arthritis Rheumatol* (2020) **72**:1990–1997. doi:10.1002/art.41422

39. Novelli L, Raimondi F, Ghirardi A, Pellegrini D, Capodanno D, Sotgiu G, Guagliumi G, Senni M, Russo FM, Lorini FL, et al. At the peak of COVID-19 age and disease severity but not comorbidities are predictors of mortality: COVID-19 burden in Bergamo, Italy. *Panminerva Med* (2021) **63**:51–61. doi:10.23736/S0031-0808.20.04063-X

40. Pan C, Chen L, Lu C, Zhang W, Xia J-A, Sklar MC, Du B, Brochard L, Qiu H. Lung Recruitability in COVID-19-associated Acute Respiratory Distress Syndrome: A Single-Center Observational Study. *Am J Respir Crit Care Med* (2020) **201**:1294–1297. doi:10.1164/rccm.202003-0527LE

41. Peng M, Liu X, Li J, Ren D, Liu Y, Meng X, Lyu Y, Chen R, Yu B, Zhong W. Successful management of seven cases of critical COVID-19 with early noninvasive-invasive

sequential ventilation algorithm and bundle pharmacotherapy. *Front Med* (2020) **14**:674–680. doi:10.1007/s11684-020-0796-3

42. Plotnikow GA, Matesa A, Nadur JM, Alonso M, Nuñez I I, Vergara G, Alfageme MJ, Vitale A, Gil M, Kinzler V, et al. Characteristics and outcomes of patients infected with nCoV19 requiring invasive mechanical ventilation in Argentina. *Rev Bras Ter Intensiva* (2020) **32**:348–353. doi:10.5935/0103-507X.20200062

43. Radnis C, Qiu S, Jhaveri M, Da Silva I, Szewka A, Koffman L. Radiographic and clinical neurologic manifestations of COVID-19 related hypoxemia. *J Neurol Sci* (2020) **418**:117119. doi:10.1016/j.jns.2020.117119

44. Riker RR, May TL, Fraser GL, Gagnon DJ, Bandara M, Zemrak WR, Seder DB. Heparin-induced thrombocytopenia with thrombosis in COVID-19 adult respiratory distress syndrome. *Res Pract Thromb Haemost* (2020) **4**:936–941. doi:10.1002/rth2.12390

45. Rizo-Téllez SA, Méndez-García LA, Flores-Rebollo C, Alba-Flores F, Alcántara-Suárez R, Manjarrez-Reyna AN, Baltazar-López N, Hernández-Guzmán VA, León-Pedroza JI, Zapata-Arenas R, et al. The Neutrophil-to-Monocyte Ratio and Lymphocyte-to-Neutrophil Ratio at Admission Predict In-Hospital Mortality in Mexican Patients with Severe SARS-CoV-2 Infection (Covid-19). *Microorganisms* (2020) **8**: doi:10.3390/microorganisms8101560

46. Sakr Y, Giovini M, Leone M, Pizzilli G, Kortgen A, Bauer M, Tonetti T, Duclos G, Zieleskiewicz L, Buschbeck S, et al. The clinical spectrum of pulmonary thromboembolism in patients with coronavirus disease-2019 (COVID-19) pneumonia: A European case series. *J Crit Care* (2021) **61**:39–44. doi:10.1016/j.jcrc.2020.09.021

47. Schaefer I-M, Padera RF, Solomon IH, Kanjilal S, Hammer MM, Hornick JL, Sholl LM. In situ detection of SARS-CoV-2 in lungs and airways of patients with COVID-19. *Mod Pathol* (2020) **33**:2104–2114. doi:10.1038/s41379-020-0595-z

48. Shen C, Wang Z, Zhao F, Yang Y, Li J, Yuan J, Wang F, Li D, Yang M, Xing L, et al. Treatment of 5 Critically Ill Patients With COVID-19 With Convalescent Plasma. *JAMA* (2020) **323**:1582–1589. doi:10.1001/jama.2020.4783

49. Singh S, Chakravarty T, Chen P, Akhmerov A, Falk J, Friedman O, Zaman T, Ebinger JE, Gheorghiu M, Marbán L, et al. Allogeneic cardiosphere-derived cells (CAP-1002) in critically ill COVID-19 patients: compassionate-use case series. *Basic Res Cardiol* (2020) **115**:36. doi:10.1007/s00395-020-0795-1

50. So C, Ro S, Murakami M, Imai R, Jinta T. High-dose, short-term corticosteroids for ARDS caused by COVID-19: a case series. *Respirol Case Rep* (2020) **8**:e00596. doi:10.1002/rcr2.596

51. Søvik S, Bådstøløkken PM, Sørensen V, Myhre PL, Prebensen C, Omland T, Berdal J-E. A single-centre, prospective cohort study of COVID-19 patients admitted to ICU for mechanical ventilatory support. *Acta Anaesthesiol Scand* (2021) **65**:351–359.

doi:10.1111/aas.13726

52. Stony Brook COVID-19 Research Consortium. Geospatial Distribution and Predictors of Mortality in Hospitalized Patients With COVID-19: A Cohort Study. *Open Forum Infect Dis* (2020) **7**:ofaa436. doi:10.1093/ofid/ofaa436

53. Wali A, Rizzo V, Bille A, Routledge T, Chambers AJ. Pneumomediastinum following intubation in COVID-19 patients: a case series. *Anaesthesia* (2020) **75**:1076–1081. doi:10.1111/anae.15113

54. Wang Y, Lu X, Li Y, Chen H, Chen T, Su N, Huang F, Zhou J, Zhang B, Yan F, et al. Clinical Course and Outcomes of 344 Intensive Care Patients with COVID-19. *Am J Respir Crit Care Med* (2020) **201**:1430–1434. doi:10.1164/rccm.202003-0736LE

55. Wang X-H, Duan J, Han X, Liu X, Zhou J, Wang X, Zhu L, Mou H, Guo S. High incidence and mortality of pneumothorax in critically Ill patients with COVID-19. *Heart Lung* (2021) **50**:37–43. doi:10.1016/j.hrtlng.2020.10.002

56. Weiskopf D, Schmitz KS, Raadsen MP, Grifoni A, Okba NMA, Endeman H, van den Akker JPC, Molenkamp R, Koopmans MPG, van Gorp ECM, et al. Phenotype and kinetics of SARS-CoV-2-specific T cells in COVID-19 patients with acute respiratory distress syndrome. *Sci Immunol* (2020) **5**: doi:10.1126/sciimmunol.abd2071

57. Wilk AJ, Rustagi A, Zhao NQ, Roque J, Martínez-Colón GJ, McKechnie JL, Ivison GT, Ranganath T, Vergara R, Hollis T, et al. A single-cell atlas of the peripheral immune response in patients with severe COVID-19. *Nat Med* (2020) **26**:1070–1076. doi:10.1038/s41591-020-0944-y

58. Zhang L, Li J, Zhou M, Chen Z. Summary of 20 tracheal intubation by anesthesiologists for patients with severe COVID-19 pneumonia: retrospective case series. *J Anesth* (2020) **34**:599–606. doi:10.1007/s00540-020-02778-8

59. Ziehr DR, Alladina J, Petri CR, Maley JH, Moskowitz A, Medoff BD, Hibbert KA, Thompson BT, Hardin CC. Respiratory Pathophysiology of Mechanically Ventilated Patients with COVID-19: A Cohort Study. *Am J Respir Crit Care Med* (2020) **201**:1560–1564. doi:10.1164/rccm.202004-1163LE

60. Akhtar W, Olusanya O, Baladia MM, Young H, Shah S. SARS-CoV-2 and ECMO: early results and experience. *Indian J Thorac Cardiovasc Surg* (2020)1–8. doi:10.1007/s12055-020-01084-y

61. Alnababteh M, Hashmi MD, Vedantam K, Chopra R, Kohli A, Hayat F, Kriner E, Molina E, Pratt A, Oweis E, et al. Extracorporeal membrane oxygenation for COVID-19 induced hypoxia: Single-center study. *Perfusion* (2020)267659120963885. doi:10.1177/0267659120963885

62. Beyls C, Huette P, Abou-Arab O, Berna P, Mahjoub Y. Extracorporeal membrane oxygenation for COVID-19-associated severe acute respiratory distress syndrome and risk

of thrombosis. *Br J Anaesth* (2020) **125**:e260–e262. doi:10.1016/j.bja.2020.04.079

63. Charlton M, Dashey S, Stubbs A, Lai FY, Bird PW, Badhwar V, Tang JW. Comparing SARS-CoV-2 and influenza A(H1N1)pdm09-infected patients requiring ECMO - A single-centre, retrospective observational cohort experience. *J Infect* (2021) **82**:84–123. doi:10.1016/j.jinf.2020.11.003

64. Falcoz P-E, Monnier A, Puyraveau M, Perrier S, Ludes P-O, Olland A, Mertes P-M, Schneider F, Helms J, Meziani F. Extracorporeal Membrane Oxygenation for Critically Ill Patients with COVID-19-related Acute Respiratory Distress Syndrome: Worth the Effort? *Am J Respir Crit Care Med* (2020) **202**:460–463. doi:10.1164/rccm.202004-1370LE

65. Goursaud S, Descamps R, Daubin C, du Cheyron D, Valette X. Corticosteroid use in selected patients with severe acute respiratory distress syndrome related to COVID-19. *J Infect* (2020) **81**:e89–e90. doi:10.1016/j.jinf.2020.05.023

66. Guihaire J, Owyang CG, Madhok J, Laverdure F, Gaillard M, Girault A, Lebreton G, Mercier O. Specific Considerations for Venovenous Extracorporeal Membrane Oxygenation During Coronavirus Disease 2019 Pandemic. *ASAIO J* (2020) **66**:1069–1072. doi:10.1097/MAT.0000000000001251

67. Guo Z, Sun L, Li B, Tian R, Zhang X, Zhang Z, Clifford SP, Liu Y, Huang J, Li X. Anticoagulation Management in Severe Coronavirus Disease 2019 Patients on Extracorporeal Membrane Oxygenation. *J Cardiothorac Vasc Anesth* (2021) **35**:389–397. doi:10.1053/j.jvca.2020.08.067

68. Heman-Ackah SM, Su YS, Spadola M, Petrov D, Chen HI, Schuster J, Lucas T. Neurologically Devastating Intraparenchymal Hemorrhage in COVID-19 Patients on Extracorporeal Membrane Oxygenation: A Case Series. *Neurosurgery* (2020) **87**:E147–E151. doi:10.1093/neuros/nyaa198

69. Huette P, Beyls C, Guilbart M, Coquet A, Berna P, Haye G, Roger P-A, Besserve P, Bernasinski M, Dupont H, et al. Extracorporeal membrane oxygenation for respiratory failure in COVID-19 patients: outcome and time-course of clinical and biological parameters. *Can J Anaesth* (2020) **67**:1486–1488. doi:10.1007/s12630-020-01727-z

70. Jäckel M, Rilinger J, Lang CN, Zotzmann V, Kaier K, Stachon P, Biever PM, Wengenmayer T, Duerschmied D, Bode C, et al. Outcome of acute respiratory distress syndrome requiring extracorporeal membrane oxygenation in Covid-19 or influenza: A single-center registry study. *Artif Organs* (2021) **45**:593–601. doi:10.1111/aor.13865

71. Jacobs JP, Stammers AH, St Louis J, Hayanga JWA, Firstenberg MS, Mongero LB, Tesdahl EA, Rajagopal K, Cheema FH, Coley T, et al. Extracorporeal Membrane Oxygenation in the Treatment of Severe Pulmonary and Cardiac Compromise in Coronavirus Disease 2019: Experience with 32 Patients. *ASAIO J* (2020) **66**:722–730. doi:10.1097/MAT.0000000000001185

72. Kon ZN, Smith DE, Chang SH, Goldenberg RM, Angel LF, Carillo JA, Geraci TC, Cerfolio

RJ, Montgomery RA, Moazami N, et al. Extracorporeal Membrane Oxygenation Support in Severe COVID-19. *Ann Thorac Surg* (2021) **111**:537–543. doi:10.1016/j.athoracsur.2020.07.002

73. Le Breton C, Besset S, Freita-Ramos S, Amouretti M, Billiet PA, Dao M, Dumont LM, Federici L, Gaborieau B, Longrois D, et al. Extracorporeal membrane oxygenation for refractory COVID-19 acute respiratory distress syndrome. *J Crit Care* (2020) **60**:10–12. doi:10.1016/j.jcrc.2020.07.013

74. Li X, Guo Z, Li B, Zhang X, Tian R, Wu W, Zhang Z, Lu Y, Chen N, Clifford SP, et al. Extracorporeal Membrane Oxygenation for Coronavirus Disease 2019 in Shanghai, China. *ASAIO J* (2020) **66**:475–481. doi:10.1097/MAT.0000000000001172

75. Liu J, Dong Y-Q, Yin J, He G, Wu X, Li J, Qiu Y, He X. Critically ill patients with COVID-19 with ECMO and artificial liver plasma exchange: A retrospective study. *Medicine* (2020) **99**:e21012. doi:10.1097/MD.0000000000021012

76. Loforte A, Dal Checco E, Gliozzi G, Benedetto M, Cavalli GG, Mariani C, Piccone G, Agulli M, Pacini D, Baiocchi M. Veno-venous Extracorporeal Membrane Oxygenation Support in COVID-19 Respiratory Distress Syndrome: Initial Experience. *ASAIO J* (2020) **66**:734–738. doi:10.1097/MAT.0000000000001198

77. Matsunaga N, Hayakawa K, Terada M, Ohtsu H, Asai Y, Tsuzuki S, Suzuki S, Toyoda A, Suzuki K, Endo M, et al. Clinical epidemiology of hospitalized patients with COVID-19 in Japan: Report of the COVID-19 REGISTRY JAPAN. *Clin Infect Dis* (2020) doi:10.1093/cid/ciaa1470

78. Miike S, Sakamoto N, Washino T, Kosaka A, Kuwahara Y, Ishida T, Hikone M, Oyabu T, Kojima H, Iwabuchi S, et al. Critically ill patients with COVID-19 in Tokyo, Japan: A single-center case series. *J Infect Chemother* (2021) **27**:291–295. doi:10.1016/j.jiac.2020.10.019

79. Mustafa AK, Alexander PJ, Joshi DJ, Tabachnick DR, Cross CA, Pappas PS, Tatooles AJ. Extracorporeal Membrane Oxygenation for Patients With COVID-19 in Severe Respiratory Failure. *JAMA Surg* (2020) **155**:990–992. doi:10.1001/jamasurg.2020.3950

80. Osho AA, Moonsamy P, Hibbert KA, Shelton KT, Trahanas JM, Attia RQ, Bloom JP, Onwugbufor MT, D'Alessandro DA, Villavicencio MA, et al. Veno-venous Extracorporeal Membrane Oxygenation for Respiratory Failure in COVID-19 Patients: Early Experience From a Major Academic Medical Center in North America. *Ann Surg* (2020) **272**:e75–e78. doi:10.1097/SLA.0000000000004084

81. Ronit A, Berg RMG, Bay JT, Haugaard AK, Ahlström MG, Burgdorf KS, Ullum H, Rørvig SB, Tjelle K, Foss NB, et al. Compartmental immunophenotyping in COVID-19 ARDS: A case series. *J Allergy Clin Immunol* (2021) **147**:81–91. doi:10.1016/j.jaci.2020.09.009

82. Schmidt M, Hajage D, Lebreton G, Monsel A, Voiriot G, Levy D, Baron E, Beurton A, Chommeloux J, Meng P, et al. Extracorporeal membrane oxygenation for severe acute

respiratory distress syndrome associated with COVID-19: a retrospective cohort study. *Lancet Respir Med* (2020) **8**:1121–1131. doi:10.1016/S2213-2600(20)30328-3

83. Shih E, DiMaio JM, Squiers JJ, Banwait JK, Meyer DM, George TJ, Schwartz GS, Baylor Scott & White ECMO for COVID Group. Venovenous extracorporeal membrane oxygenation for patients with refractory coronavirus disease 2019 (COVID-19): Multicenter experience of referral hospitals in a large health care system. *J Thorac Cardiovasc Surg* (2020) doi:10.1016/j.jtcvs.2020.11.073

84. Sultan I, Habertheuer A, Usman AA, Kilic A, Gnall E, Friscia ME, Zubkus D, Hirose H, Sanchez P, Okusanya O, et al. The role of extracorporeal life support for patients with COVID-19: Preliminary results from a statewide experience. *J Card Surg* (2020) **35**:1410–1413. doi:10.1111/jocs.14583

85. Usman AA, Han J, Acker A, Olia SE, Bermudez C, Cucchiara B, Mikkelsen ME, Wald J, Mackay E, Szeto W, et al. A Case Series of Devastating Intracranial Hemorrhage During Venovenous Extracorporeal Membrane Oxygenation for COVID-19. *J Cardiothorac Vasc Anesth* (2020) **34**:3006–3012. doi:10.1053/j.jvca.2020.07.063

86. Xuan W, Chen C, Jiang X, Zhang X, Zhu H, Zhang S, Yu W, Peng Z, Su D. Clinical characteristics and outcomes of five critical COVID-19 patients treated with extracorporeal membrane oxygenation in Leishenshan Hospital in Wuhan. *J Clin Anesth* (2020) **67**:110033. doi:10.1016/j.jclinane.2020.110033

87. Xu J, Xie J, Du B, Tong Z, Qiu H, Bagshaw SM. Clinical Characteristics and Outcomes of Patients With Severe COVID-19 Induced Acute Kidney Injury. *J Intensive Care Med* (2021) **36**:319–326. doi:10.1177/0885066620970858

88. Yang X, Cai S, Luo Y, Zhu F, Hu M, Zhao Y, Zheng R, Li X, Hu B, Peng Z. Extracorporeal Membrane Oxygenation for Coronavirus Disease 2019-Induced Acute Respiratory Distress Syndrome: A Multicenter Descriptive Study. *Crit Care Med* (2020) **48**:1289–1295. doi:10.1097/CCM.0000000000004447

89. Zayat R, Kalverkamp S, Grottke O, Durak K, Dreher M, Autschbach R, Marx G, Marx N, Spillner J, Kersten A. Role of extracorporeal membrane oxygenation in critically Ill COVID-19 patients and predictors of mortality. *Artif Organs* (2021) **45**:E158–E170. doi:10.1111/aor.13873

90. Zeng Y, Cai Z, Xianyu Y, Yang BX, Song T, Yan Q. Prognosis when using extracorporeal membrane oxygenation (ECMO) for critically ill COVID-19 patients in China: a retrospective case series. *Crit Care* (2020) **24**:148. doi:10.1186/s13054-020-2840-8

91. Zeng J-H, Wu W-B, Qu J-X, Wang Y, Dong C-F, Luo Y-F, Zhou D, Feng W-X, Feng C. Cardiac manifestations of COVID-19 in Shenzhen, China. *Infection* (2020) **48**:861–870. doi:10.1007/s15010-020-01473-w

92. Zhang J, Merrick B, Correa GL, Camporota L, Retter A, Doyle A, Glover GW, Sherren PB, Tricklebank SJ, Agarwal S, et al. Veno-venous extracorporeal membrane oxygenation in

coronavirus disease 2019: a case series. *ERJ Open Res* (2020) **6**: doi:10.1183/23120541.00463-2020

93. Zhang Q, Shen J, Chen L, Li S, Zhang W, Jiang C, Ma H, Lin L, Zheng X, Zhao Y. Timing of invasive mechanic ventilation in critically ill patients with coronavirus disease 2019. *J Trauma Acute Care Surg* (2020) **89**:1092–1098. doi:10.1097/TA.0000000000002939

94. Zheng Y, Sun L-J, Xu M, Pan J, Zhang Y-T, Fang X-L, Fang Q, Cai H-L. Clinical characteristics of 34 COVID-19 patients admitted to intensive care unit in Hangzhou, China. *J Zhejiang Univ Sci B* (2020) **21**:378–387. doi:10.1631/jzus.B2000174
